# Dual-targeted peptide-conjugated multifunctional fluorescent probe with AIEgen for efficient nucleus-specific imaging and long-term tracing of cancer cells†
†Electronic supplementary information (ESI) available: Experimental procedures, structural characterization data, and additional figures and scheme. See DOI: 10.1039/c7sc00402h



**DOI:** 10.1039/c7sc00402h

**Published:** 2017-04-19

**Authors:** Yong Cheng, Chunli Sun, Xiaowen Ou, Bifeng Liu, Xiaoding Lou, Fan Xia

**Affiliations:** a Hubei Key Laboratory of Bioinorganic Chemistry & Materia Medica , School of Chemistry and Chemical Engineering , Huazhong University of Science and Technology , Wuhan 430074 , P. R. China . Email: louxiaoding@hust.edu.cn ; Email: xiafan@hust.edu.cn; b National Engineering Research Center for Nanomedicine , Department of Biomedical Engineering , College of Life Science and Technology , Huazhong University of Science and Technology , Wuhan 430074 , P. R. China

## Abstract

Precisely targeted transportation of a long-term tracing regent to a nucleus with low toxicity is one of the most challenging concerns in revealing cancer cell behaviors. Here, we report a dual-targeted peptide-conjugated multifunctional fluorescent probe (cNGR-CPP-NLS-RGD-PyTPE, TCNTP) with aggregation-induced emission (AIE) characteristic, for efficient nucleus-specific imaging and long-term and low-toxicity tracing of cancer cells. TCNTP mainly consists of two components: one is a functionalized combinatorial peptide (TCNT) containing two targeted peptides (cNGR and RGD), a cell-penetrating peptide (CPP) and a nuclear localization signal (NLS), which can specifically bind to a cell surface and effectively enter into the nucleus; the other one is an AIE-active tetraphenylethene derivative (PyTPE, a typical AIEgen) as fluorescence imaging reagent. In the presence of aminopeptidase N (CD13) and integrin α_v_β_3_, TCNTP can specifically bind to both of them using cNGR and RGD, respectively, lighting up its yellow fluorescence. Because it contains CPP, TCNTP can be effectively integrated into the cytoplasm, and then be delivered into the nucleus with the help of NLS. TCNTP exhibited strong fluorescence in the nucleus of CD13 and integrin α_v_β_3_ overexpression cells due to the specific targeting ability, efficient transport capacity and AIE characteristic in a more crowded space. Furthermore, TCNTP can be applied for long-term tracing in living cells, scarcely affecting normal cells with negligible toxicity in more than ten passages.

## Abstract 2

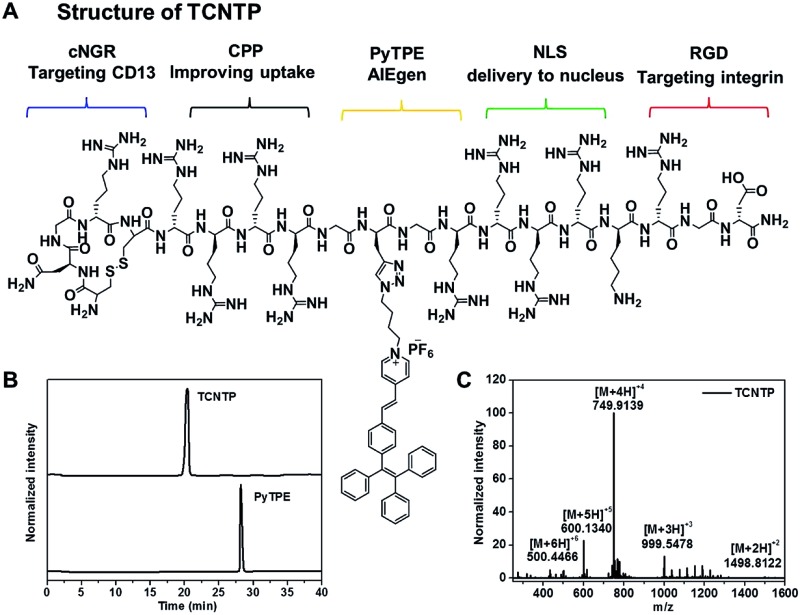
Precisely targeted transportation of a long-term tracing regent to a nucleus with low toxicity is one of the most challenging concerns in revealing cancer cell behaviors.

## Introduction

Efficient targeted delivery and long-term imaging of cancer cells remain very imperative issues for early diagnosis,^1^ accurate treatment,^2^ therapeutic monitoring^3^ as well as understanding of tumor invasion and metastasis.^4^ With a more profound understanding of cancer cells and their microenvironment,^5^ targeted delivery described as ‘magic bullet’ or ‘guided missile’ is being pursued to deliver cargoes more precisely to cancer cells rather than the traditional method based on the enhanced permeability and retention effect.^6^ Thus far, various approaches for targeted delivery strategies have been widely developed and even applied to clinical use, including molecular-targeted delivery and ligand-targeted delivery.^7^ In particular, ligand-targeted delivery can apparently improve the cell uptake of the modified cargoes through specific binding to receptors that are overexpressed on the cancer cell surface. To date, very many targeted ligands have been discovered for use in targeted delivery, such as peptides,^8^ antibodies^9^ and aptamers.^10^ Therefore, imaging reagents could be located more precisely at tumor sites *via* ligand-targeted delivery to improve tissue selectivity and imaging sensitivity.^11^ However, it is worthwhile to note that there are still two main challenges in constructing a multifunctional probe. Firstly, how to precisely transport to subcellular organelles, which means the probe needs to be precisely located to the target cell, and then cross over the cell membrane and the organelle membrane successively. Secondly, how to conduct long-term tracing for the organelle, which means the imaging agent in the probe should be stable and of low toxicity.^12^

Taking biological compatibility and availability into account, peptide-conjugated targeted delivery systems are endowed with great advantages and have made significant progress in targeted delivery,^6,7,11^ such as NGR (Asn–Gly–Arg)- and RGD (Arg–Gly–Asp)-based targeted delivery of drugs and imaging agents. On the other hand, cell-penetrating peptide (CPP) and nuclear localization signal (NLS) have attracted significant attention and been extensively applied in nucleocytoplasmic transport of biological material.^13^ However, in view of the complex system of living organisms with numerous biological barriers,^14^ sole targeted-ligand or mono-functional imaging system is oversimplified and restricted for efficient and precise location and long-term imaging in living cells.^15^ Although some multi-functional strategies have been established,^16^ the intricate and multiple steps of chemical modification and purification are too difficult to achieve optimal outcomes. Thanks to Fmoc-based solid phase peptide synthesis and combinatorial protein engineering,^17^ different kinds of targeted ligands and functional components are being progressively combined and used for multi-functional delivery, which make possible efficient cell nucleus imaging.^18^

For the better understanding of the complicated behavior of cancers, various imaging techniques have been coupled with multi-functional delivery systems, for instance, magnetic resonance imaging, positron emission tomography and fluorescence imaging. Particularly, fluorescence imaging as a feasible method has been widely investigated.^19^ It can be used to visualize the activity of biological processes that influence the behavior of cancers or responsiveness to therapeutic drugs in a simple, convenient and diversified way. However, conventional fluorescence imaging is mainly based on the aggregation-caused quenching effect which is not suitable for long-term tracking in the cell nucleus.^20^ Fortunately, Tang's group described in 2001 an unusual photophysical phenomenon named the aggregation-induced emission (AIE) effect.^21^ Propeller-shaped AIEgens are non-emissive when dispersed, but emit intensely in the aggregated state. Owing to this distinct characteristic, AIEgens might aggregate in more crowded space, such as the cell nucleus, and emit strong fluorescence.^22^ What is more, these kinds of fluorescence imaging reagents show superior photostability and excellent bioapplications and thus could be utilized for long-term and low-toxicity tracing of cancer cells.^23^

**Scheme 1:**
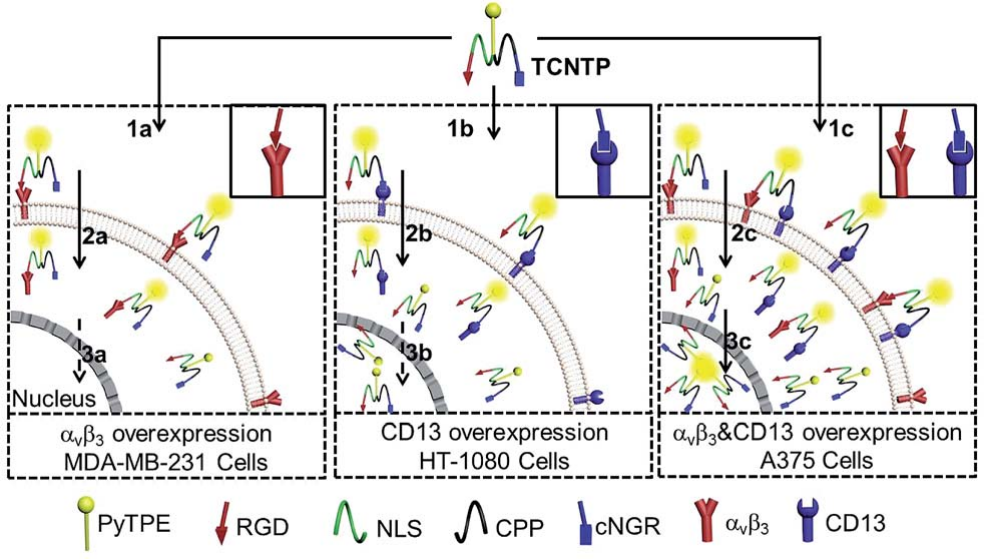
Schematic illustration of TCNTP as a fluorescence light-up nuclear penetrating dye in integrin α_v_β_3_ and CD13 overexpression living cells. The integrin α_v_β_3_ and CD13 overexpression cells would bind more TCNTP in cytomembrane which could then enter into the nucleus easily with further incubation.

**Fig. 1:**
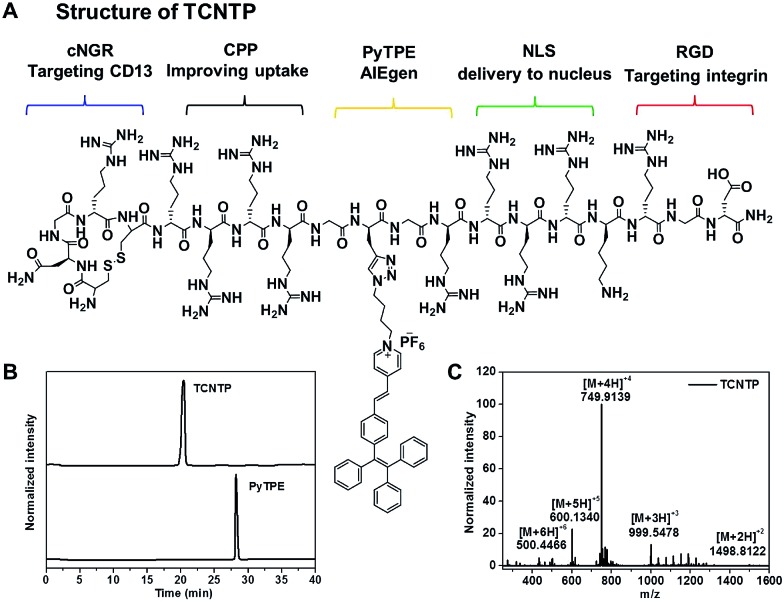
(A) The chemical structures of TCNTP. (B) High performance liquid chromatography (HPLC) results of TCNTP, and PyTPE. (C) High resolution mass spectrum (HRMS) of TCNTP.

By creatively combining a multi-functional delivery system and AIEgen, we synthesized a dual-targeted peptide-conjugated fluorescent probe (cNGR-CPP-NLS-RGD-PyTPE, TCNTP) for efficient nucleus-specific imaging and long-term and low-toxicity tracing of cancer cells (Scheme 1). TCNTP contains two parts, a multi-functional peptide delivery system (TCNT, CNGRCRRRRG(Pra)GRRRRKRGD-NH_2_) and a fluorescence imaging reagent (PyTPE) (Fig. 1A and Scheme S1†). There are four segments in TCNT which contribute to the whole delivery process. (1) cNGR (CNGRC), a targeted and cyclic peptide motif of minimal cell attachment sequence with high affinity to aminopeptidase N (CD13), which also initiated a comprehensive study for targeted delivery systems.^24^ (2) CPP (RRRR), a cell-penetrating peptide, which transports cargoes into cells in an efficient way and avoids too much nonspecific binding.^25^ (3) NLS (RRRRK), a nuclear localization signal sequence, which could be transported into the nucleus by importin or nucleic acid.^13,26^ It is worth noting that these arginine-rich sequences could act as parts of CPP on the outside of the cells. (4) RGD (RGD-NH_2_), another targeted peptide motif of minimal cell attachment sequence, having high affinity to integrin α_v_β_3_ which are highly expressed in specific tumor cells.^27^ Moreover, the cyclic peptide exhibited higher affinity toward specific recognition sites compared with the linear peptide.^28^ Finally, a propargyl modified in triglycine sequence could link TCNT with PyTPE using copper-catalyzed azide–alkyne click reaction.

In the presence of integrin α_v_β_3_ and CD13, TCNTP can specifically bind to both of them using RGD and cNGR, respectively. After attaching to the recognition sites of the cell surface, the emission of TCNTP could be switched on. In the following, the guanidinium-rich CPP could enhance the charge and solubility of TCNTP to adapt to cell environments and efficiently pass through cell membranes or endo-lysosome.^29^ Furthermore, the NLS contributed to transportation of TCNTP into the nucleus, which was beneficial to tracking the whole process of molecules into cells and nucleus. As a result, TCNTP exhibited strong fluorescence in the nucleus of integrin α_v_β_3_ and CD13 overexpression cells due to the specific targeting ability, efficient transport capacity and AIE characteristic in more crowded space.

## Results and discussion

### Design principle of TCNTP

Inspired by the typical structure of an antibody, a multi-functional and smart delivery system could be implemented. First of all, as the antigen-binding fragments (Fab), cNRG and RGD peptide should be fixed at the end of TCNTP for strengthening the targeted efficiency and scope.^30^ In the following, CPP and NLS peptide were located in the middle part of TCNTP, which could not only improve the efficiency of transportation but also accelerate across the membrane and escape from the endo-lysosome. Specifically, if there are too many arginine-rich peptides, it would reduce the selectivity of the probe towards cancer cells. In this work, CPP is chosen with the least number of arginine units,^31^ and we dispersed ten arginine residues in TCNTP (cNGR, RGD and NLS also contain arginine residues) to balance its penetration ability and targeted binding ability. This is essential to help the probe internalize into cells as well as ensure the integrity of the peptide-based probe. However, the behavior and efficiency of the probe would be weakened if we changed the arrangement between cNGR and RGD and CPP and NLS. A propargyl modified triglycine sequence was chosen as a linker to reduce steric hindrance and afford reaction sites for click reaction. The last part is a crystallizable fragment (Fc), PyTPE, which is a fluorescent tracker and beneficial to the formation of a self-assembly structure.^32^ The fluorescence turn-on is based on the specific targeting binding of targeted peptides and the increased concentrations of PyTPE in more crowded space to increase its aggregation degree.

### Synthesis of TCNTP

Firstly, we designed TCNT and customized it which was from GL Biochem Ltd (Shanghai, China). Subsequently, PyTPE was prepared according to the procedures in our previous work.^33^ Finally, the coupling between azide-functionalized TCNT and azide-functionalized PyTPE through a “copper-catalyzed azide–alkyne click” reaction afforded TCNTP in a simple and effective way (Scheme S1†). The products were characterized thoroughly by high performance liquid chromatography (HPLC), nuclear magnetic resonance spectra and high resolution mass spectra (HRMS) to confirm their chemical structure and purity (Fig. S1–S4†). As can be seen in Fig. 1B and C, the appearance time of TCNTP was earlier than that of PyTPE, and we observed a strong peak (at 999.5478) which was attributed to [M + 3H]^3+^ ion (calculated, 999.5407); strong peak (at 749.9139), which was attributed to [M + 4H]^4+^ ion (calculated, 749.9075); strong peak (at 600.1340) which was attributed to [M + 5H]^5+^ ion (calculated, 600.1276); and strong peak (at 500.4466), which was attributed to [M + 6H]^6+^ ion (calculated, 500.2743). These results indicated that TCNTP was successfully synthesized.

### Response property in solutions

**Fig. 2:**
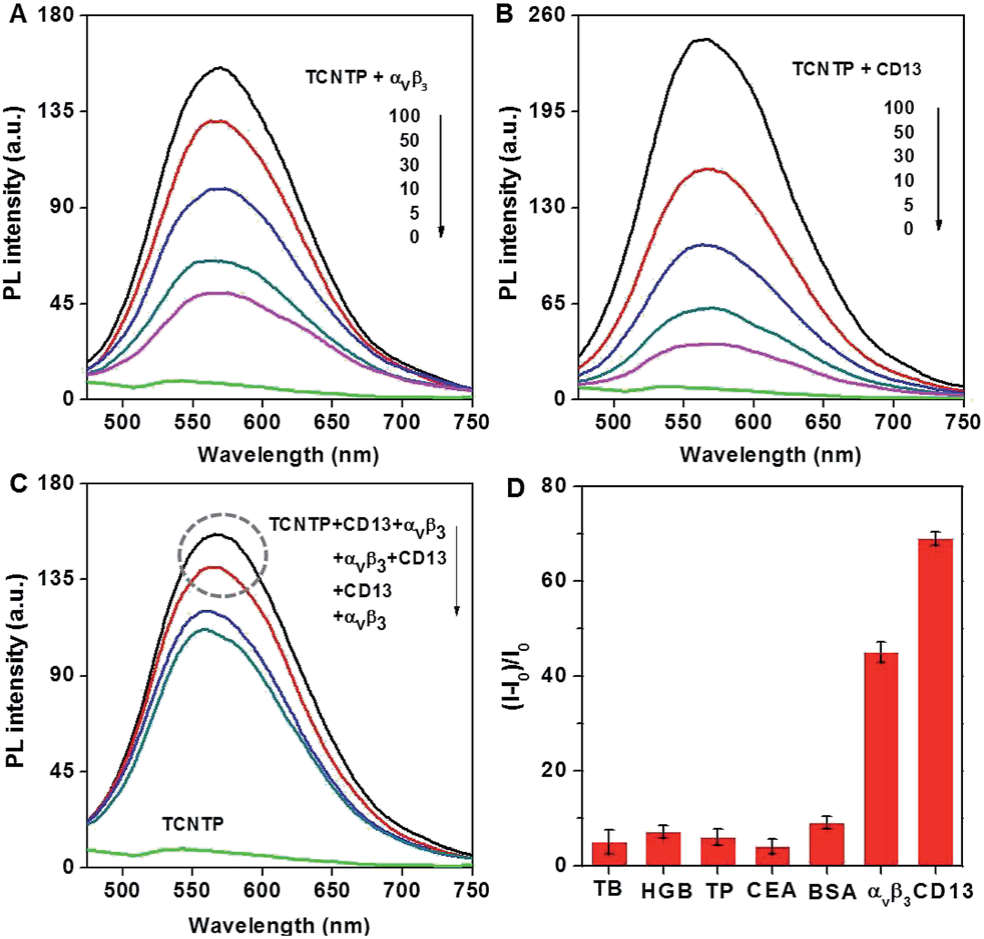
Photoluminescence (PL) spectra of TCNTP (10 μM) treated with different concentrations of (A) α_v_β_3_ (0, 5, 10, 30, 50, 100 μg mL^–1^) and (B) CD13 (0, 5, 10, 30, 50, 100 μg mL^–1^) for 30 min at 37 °C. (C) PL spectra of TCNTP (10 μM) treated with 30 μg mL^–1^ integrin α_v_β_3_ (or CD13) and then 30 μg mL^–1^ CD13 (or integrin α_v_β_3_) for 30 min at 37 °C. (D) Plot of (*I* – *I*_0_)/*I*_0_ *versus* different proteins (thrombin (TB), hemoglobin (HGB), trypsin (TP), carcinoembryonic antigen (CEA), bovine serum albumin (BSA)), where *I* and *I*_0_ are the PL intensities at analyte concentrations of 50 and 0 μg mL^–1^, respectively. *λ*_ex_ = 405 nm.

PyTPE and TCNTP showed similar absorption spectral profiles at 230–300 nm, 300–350 nm and 350–500 nm, while TCNTP showed a stronger absorption at 230–300 nm than PyTPE owing to the peptide characteristic absorption peak (Fig. S5A†). PyTPE exhibited very strong fluorescence intensity from 500 to 560 nm in a mixed solution of DMSO/water (1 : 199 v/v) at room temperature. After being modified with water-soluble peptide, the fluorescence intensity of TCNTP decreased obviously due to its good water solubility. By increasing the dye concentration from 10 μM to 100 μM, the fluorescence intensity increased, suggesting that TCNTP started to form micelles (Fig. S5B†). Thus, 10 μM was chosen as an optimal concentration for this strategy. To validate the specific binding ability to integrin α_v_β_3_ and CD13, TCNTP was incubated with different concentrations of integrin α_v_β_3_ and CD13. As can been seen from Fig. 2A, the fluorescence intensity of TCNTP was gradually enhanced with the increase of integrin α_v_β_3_ concentration. Such a phenomenon was also observed when TCNTP was incubated with increasing CD13 concentration (Fig. 2B). Moreover, CD13 led to much stronger TCNTP fluorescence intensity than integrin α_v_β_3_.

To further prove TCNTP was more sensitive to CD13 than integrin α_v_β_3_, programmed tests were performed. 10 μM TCNTP was incubated with 30 μg mL^–1^ integrin α_v_β_3_ (or CD13) and then 30 μg mL^–1^ CD13 (or integrin α_v_β_3_) for 30 min at 37 °C, the fluorescence changes being recorded after incubation. As shown in Fig. 2C, CD13 had a greater influence on fluorescence intensity changes than integrin α_v_β_3_. Finally, TCNTP was treated under identical conditions with several commercial proteins to investigate its selectivity. The fluorescence intensity was obvious enhanced only when incubated with integrin α_v_β_3_ and CD13, and CD13 was more sensitive (Fig. 2D). All these fluorescent measurements illustrated that TCNTP was not only binding to integrin α_v_β_3_ but also to CD13 with high specificity.

### Confocal imaging of living cells incubated with TCNTP

In an effort to learn the effective targeting and nucleus imaging properties of TCNTP in living cells, three different cell lines were chosen for confocal laser scanning microscopy (CLSM) observation, namely breast cancer cells MDA-MB-231 (α_v_β_3_ overexpression cell line), fibrosarcoma cells HT-1080 (CD13 overexpression cell line) and human malignant melanoma cells A375 (α_v_β_3_ and CD13 overexpression cell line).^27,34^ HT-1080 cells and MDA-MB-231 cells are flat cells while A375 cells are fibrous cells which are easy to be distinguished. To be an effective bioprobe, TCNTP must be appropriate in mixed populations of different cell lines. MDA-MB-231 cells and A375 cells, and HT-1080 cells and A375 cells were respectively co-cultured in a mixed medium including 50% 1640 medium and 50% DMEM medium at 37 °C in a humidified atmosphere containing 5% CO_2_ to assess the targeting ability of TCNTP towards integrin α_v_β_3_ and CD13 in living cells.

**Fig. 3:**
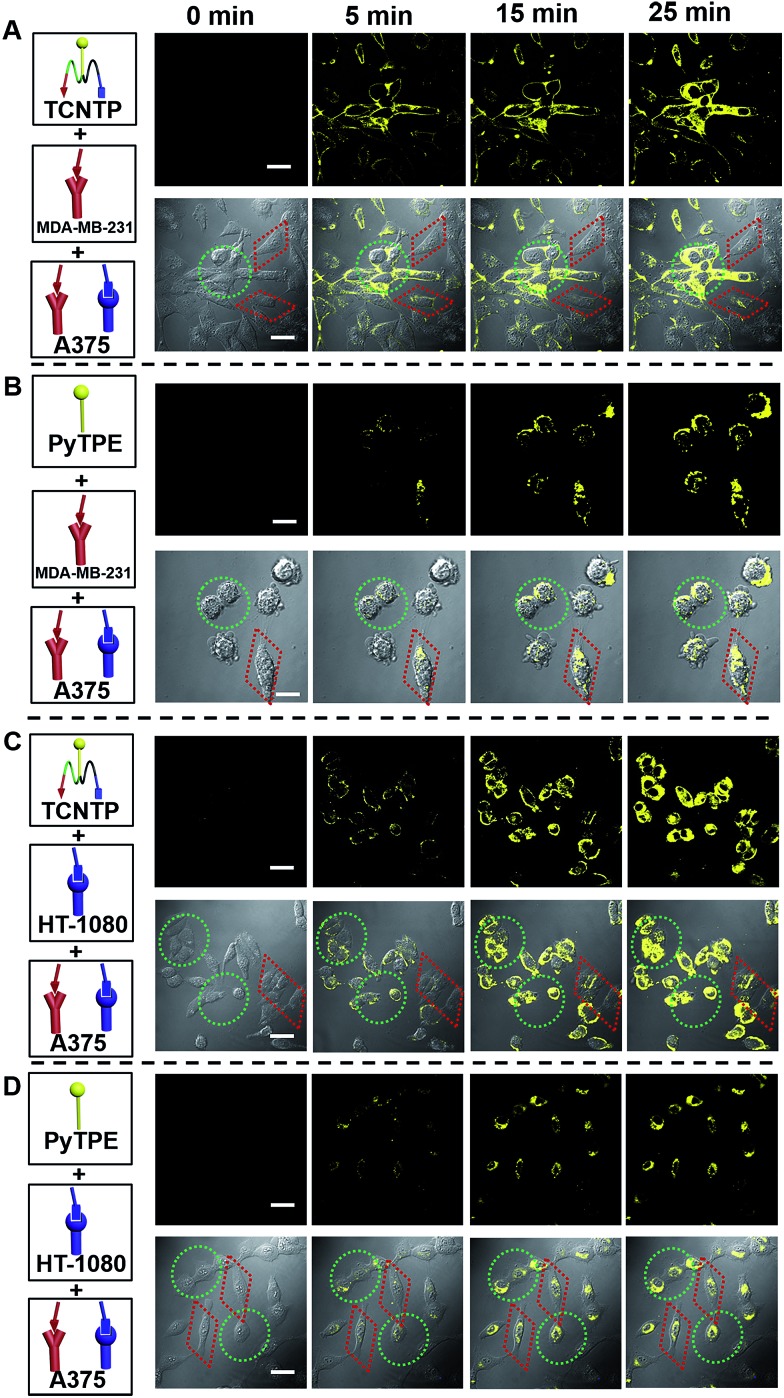
*In situ* and real-time CLSM images of co-cultured cells incubated with TCNTP and PyTPE. MDA-MB-231 cells (red square) and A375 cells (green circle) incubated with TCNTP ((A) 3.0 μM) and PyTPE ((B) 3.0 μM) for 25 min, respectively; HT-1080 cells (red square) and A375 cells (green circle) incubated with TCNTP ((C) 3.0 μM) and PyTPE ((D) 3.0 μM) for 25 min, respectively. HT-1080 cells and A375 cells incubated with TCNTP ((A) 3.0 μM) and PyTPE ((B) 3.0 μM) for 25 min, respectively. The yellow fluorescence channel: excitation wavelength, 405 nm; emission collected, 495–575 nm. The scale bar is 20 μm.

To determine the optimized concentration for cell imaging, different concentrations (1.0 μM, 3.0 μM, 5.0 μM and 10.0 μM) of TCNTP were cultured with MDA-MB-231 cells for 4 h as shown in Fig. S6.† According to the CLSM images, 3 μM concentration was enough and most suitable for cell imaging, and 10 μM probe-incubated cells were too bright to distinguish the selectivity of the probe towards different cancer cells. As a result, 3 μM is the optimum concentration for cell imaging experiments. *In situ* and real-time cell imaging experiments were performed for co-cultured cells (Fig. 3). After adding 3 μM TCNTP into MDA-MB-231 and A375 co-cultured cells, yellow fluorescence was apparently observed within 5 min in the A375 cells but not in the MDA-MB-231 cells. The yellow fluorescence of A375 cells increased with time, while being almost invisible in MDA-MB-231 cells (Fig. 3A). In addition, by reducing the concentration (1 μM) of TCNTP, we obtained similar results fir MDA-MB-231 and A375 co-cultured cells as shown in Fig. S7.† This phenomenon was also observed in HT-1080 and A375 co-cultured cells (Fig. 3C). However, the fluorescence difference was not obvious due to the higher binding capacity of cyclic targeted peptide (cNGR) compared with linear targeted peptide (RGD). In comparison, 3 μM PyTPE-incubated MDA-MB-231 and A375, and HT-1080 and A375 co-cultured cells did not exhibit strong yellow fluorescence within 5 min, and the fluorescence intensity increased slowly without selective cellular uptake (Fig. 3B and D). These results demonstrated significant differences between TCNTP and PyTPE in the quantity and efficiency of cell uptake attributed to CPP. The average fluorescence intensity of MDA-MB-231 and A375 co-cultured cells and HT-1080 and A375 co-cultured cells respectively incubated with 3.0 μM TCNTP and PyTPE for 25 min further confirmed the effects of GRD, cNGR and CPP (Fig. S8†).

**Fig. 4:**
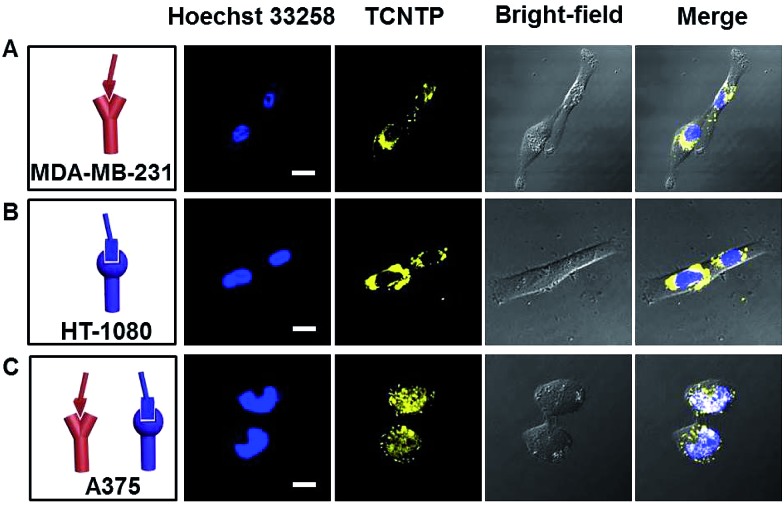
The CLSM images of MDA-MB-231 cells (A), HT-1080 cells (B) and A375 cells (C) incubated with Hoechst 33258 (3.0 μM) for 30 min and TCNTP (3.0 μM) for 4 h in cell culturing conditions. The blue fluorescence channel: excitation wavelength, 405 nm; emission collected, 425–485 nm. The yellow fluorescence channel: excitation wavelength, 405 nm; emission collected, 495–575 nm. The scale bar is 20 μm.

To investigate the nuclear localization of TCNTP, commercial dye Hoechst 33258 as an indicator, which stains the nuclei of living cells, was chosen for colocalization experiments. Three cell lines were successively incubated with 3 μM TCNTP for 4 hours and then 3 μM Hoechst 33258 for 30 min before imaging. As we can see from the CLSM image of α_v_β_3_ and CD13 overexpression A375 cells in Fig. 4C, obvious yellow fluorescence from TCNTP was observed and overlapped well with the blue fluorescence of Hoechst 33258 (Pearson correlation coefficient: 80%). By reducing the concentration to 1 μM and incubation time to 30 min of TCNTP, we obtained similar results in that only light yellow fluorescence from TNCTP was observed in the nucleus as shown in Fig. S9.† When we prolonged the incubation time (12 h), the yellow fluorescence intensity of the nucleus was enhanced with a Pearson correlation coefficient of 92% (Fig. S10 and S11†). Furthermore, yellow and blue fluorescence did not overlap with each other in single-protein overexpressed cells (MDA-MB-231 and HT-1080) shown in Fig. 4A and B, demonstrating the good selectivity of TCNTP. Additionally, three different cell lines were treated with both PyTPE and Hoechst 33258 (Fig. S12†). Owing to the positively charged electron-accepting pyridinium unit, PyTPE exhibited strong yellow fluorescence inside the cells.^33^ However, the nucleus would not be stained regardless of increasing the incubation time and concentration. This is also confirmed by three-dimensional imaging (Fig. S13–S16†). These results provide direct evidence for the ability to image a specific cell line with TCNTP. To further confirm the nucleus-specific imaging of TCNTP, a PyTPE-labelled nuclear localization signal sequence (NLS-PyTPE) was synthesized and used as a control probe. The chemical structure and HRMS data are shown in Fig. S17.† MDA-MB-231 cells, HT-1080 cells, A375 cells and MDA-MB-231 and A375 co-cultured cells were treated with 3 μM NLS-PyTPE for 4 h and 8 h in cell culturing conditions. As shown in Fig. S18,† for both culture times there was almost no fluorescence in nucleus areas. To further investigate the nuclear localization of NLS-PyTPE, Hoechst 33258 was chosen for colocalization experiments in 8 h incubation. Obvious yellow fluorescence from NLS-PyTPE was observed in all three cell lines and overlapped well with the blue fluorescence of Hoechst 33258 (Fig. S19†). These results indicated that NLS peptide alone cannot provide selectivity towards different cell lines. Target peptides RGD and cNGR play an important role in the selectivity of TCNTP.

### Long-term tracking of cancer cells

**Fig. 5:**
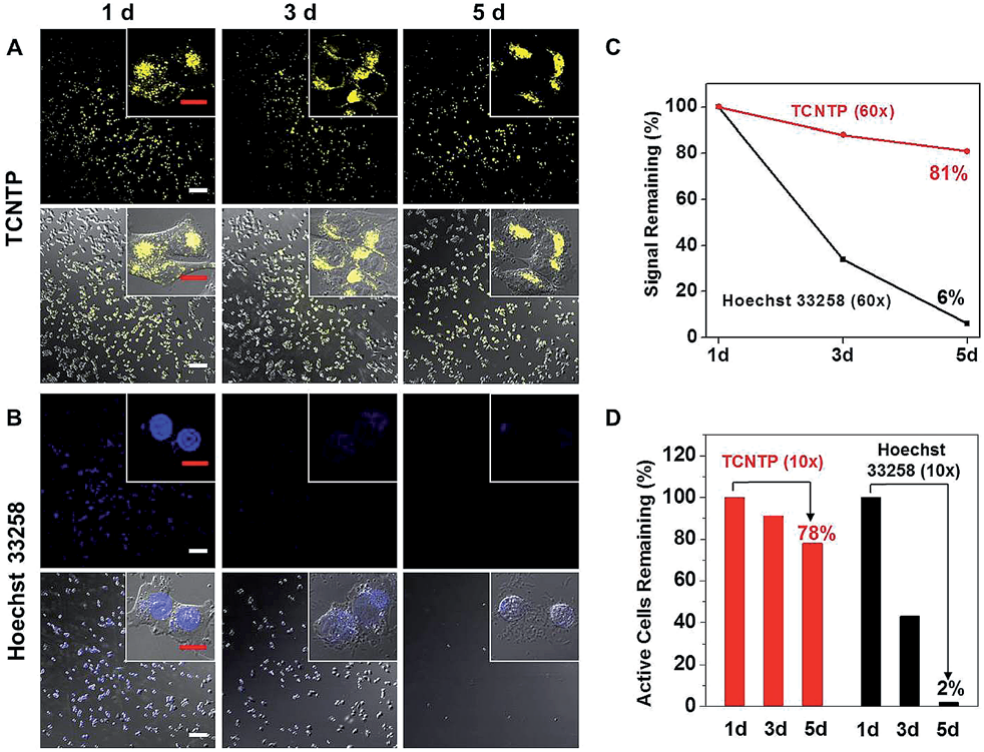
The CLSM imaging of A375 cells treated with 3 μM TCNTP (A) and 3 μM Hoechst 33258 (B) for 4 h with further incubation for 1 d, 3 d and 5 d in cell culturing conditions. (C) The average fluorescence intensity of TCNTP and Hoechst 33258 in 60× microscopic field (the insets). (D) The number of living A375 cells in 10× microscopic field. The white scale bar: 100 μm; the red scale bar: 10 μm.

Due to reverse diffusion, most conventional dyes cannot be retained in living cells for more than a few hours.^35^ Although some cell-tracker fluorescence dyes, for example Hoechst stains, can remain in viable cells for a few generations, they bind to DNA and thus interfere with DNA replication during cell division which can be cytotoxic. The harmless physical internalization processes and indelible cellular staining property suggest that TCNTP may be used for long-term tumor cell tracing. A375 cells were incubated separately with each of 3 μM TCNTP and Hoechst 33258. After 4 h incubation, the probe (or dye) was discarded and the labelled cells continued to be cultured overnight. Then the labelled cells were digested and divided into two dishes for another overnight incubation, referred to as one culture cycle. One of the dishes was treated with the next culture cycle, while the other one was used for CLSM imaging observation. During the first culture cycle (1 d), TCNTP-labelled cells showed strong yellow fluorescence in the nucleus (Fig. 5A). While the yellow fluorescence of TCNTP mainly distributed to cytoplasm for the third culture cycle (3 d) due to degradation of peptides and redistribution of the probe during cell division. After the degradation of peptides, TCNTP residues (probably TPE-Py) were unable to be delivered into the nucleus for nucleus-specific imaging. It is true that TCNTP is unstable for nucleus localization. However, it is stable for long-term cellular localization.

The cells stained by TCNTP were still visible even after the tenth culture cycle (Fig. S20†), whereas very weak fluorescence signals were detected in the Hoechst 33258-stained cells after the third culture cycle (Fig. 5B). The fluorescence signal remaining and number of remaining TCNTP-stained cells are 81% and 78%, respectively, while the percentages for commercial Hoechst 33258 are 6% and 2% after the fifth culture cycle (Fig. 5C and D). Additionally, PyTPE showed negligible toxicity to all the cell lines according to the MTT assays of cell viability, while TCNTP had slight selective toxicity due to the bioactive peptide36 and Hoechst had high toxicity due to the selective binding to minor groove of DNA37 (Fig. S21†). As a result, TCNTP could trace living cells for over 240 h, proving that it is a superb long-term and low-toxicity cell tracer. In the future, we may add non-natural amino acids in our peptide based probe to enhance its stability.

### Leak-free marking of cancer cells

The cytocompatible nature of TCNTP and its precisely targeted interaction with organelles may account for the leak-free marking inside cells. A375 cells stained by TCNTP were co-cultured with MDA-MB-231 cells or human lung fibroblast (HLF) cells (normal human cell line), in order to evaluate whether TCNTP could be utilized to discriminate between different cell lines. TCNTP could easily stain the MDA-MB-231 cells and HLF cells in monoculture experiment (Fig. S22†).

**Fig. 6:**
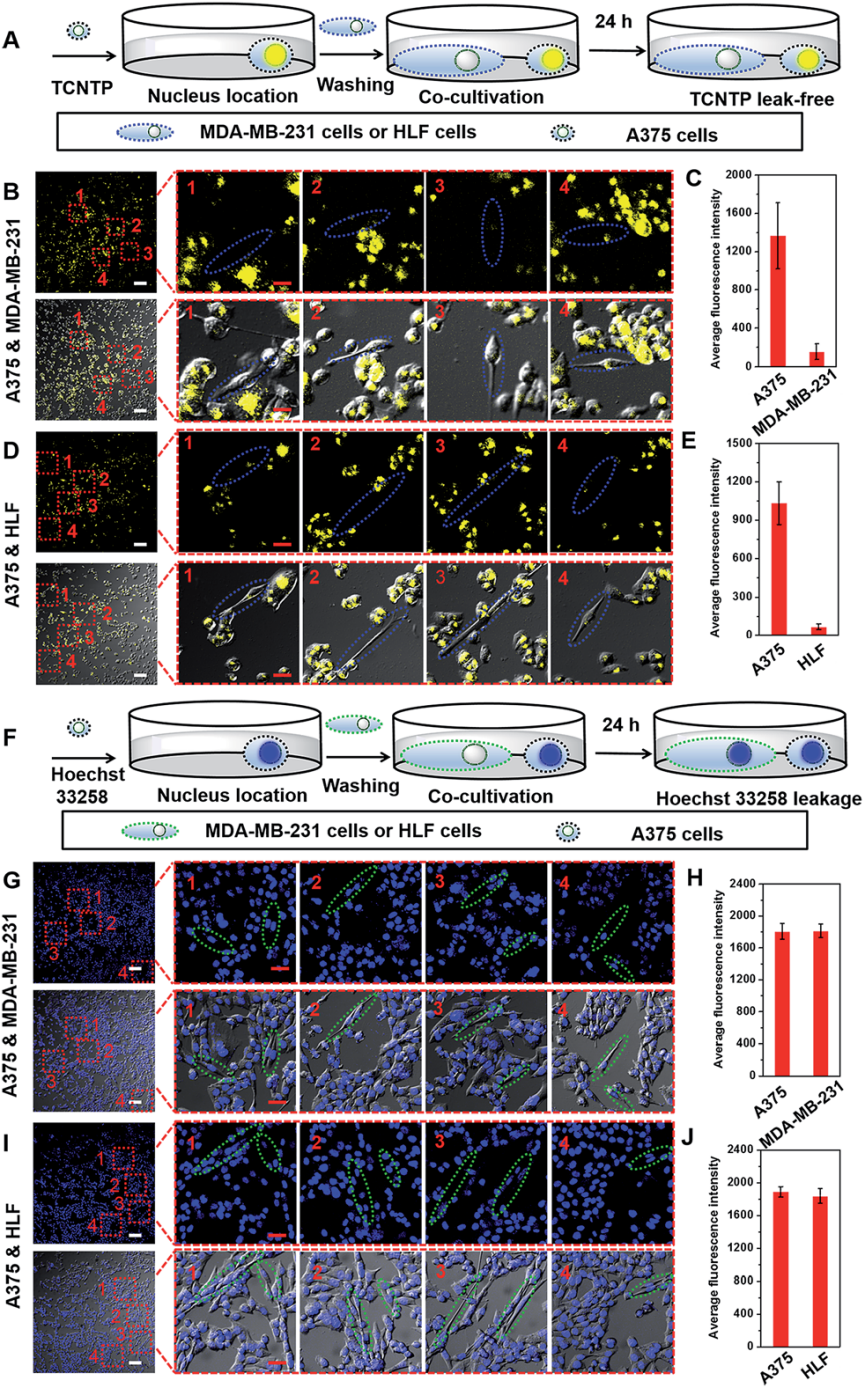
The experimental scheme of TCNTP-stained (A) or Hoechst 33258-stained (F) A375 cells co-cultured with unstained cells. The CLSM imaging of co-cultivated TCNTP-stained (B) A375 cells and unstained MDA-MB-231 cells (the blue ellipses) or (D) TCNTP-stained A375 cells and unstained HLF cells (the blue ellipses). The average fluorescence intensity of TCNTP in four partial enlarged views (C and E). The CLSM imaging of co-cultivated Hoechst 33258-stained (G) A375 cells and unstained MDA-MB-231 cells (the green ellipses) or (I) Hoechst 33258-stained A375 cells and unstained HLF cells (the green ellipses). The average fluorescence intensity of Hoechst 33258 in four partial enlarged views (H and J). The blue fluorescence channel: excitation wavelength, 405 nm; emission collected, 425–485 nm. The yellow fluorescence channel: excitation wavelength, 405 nm; emission collected, 495–575 nm. The white scale bar is 100 μm. The red scale bar is 20 μm.

However, yellow fluorescence of TCNTP remained in the A375 cells without penetrating into MDA-MB-231 cells or HLF cells after the two kinds of cells were co-cultured for 24 h, demonstrating that TCNTP was a leak-free probe (Fig. 6A–E). Under similar experimental conditions, Hoechst 33258 failed to show discrimination function because of its fast leakage (Fig. 6F–J). It is demonstrated that the TCNTP-stained A375 cells could keep admirable stability in long-time incubation and not suffer interference from other cell lines compared with Hoechst 33258. The excellent intracellular preservation of TCNTP may be used to develop novel fluorescent probes for differentiating specific cancerous cells from normal cells and for tracing diffusion, necrosis, shrinkage and suppression processes of tumor cells.

## Conclusions

In this work, a dual-targeted peptide-conjugated fluorescent probe was synthesized which contained a multi-functional peptide delivery system and fluorescence imaging reagent. Thanks to the good water solubility of the peptide, the fluorescence of TCNTP is initially quenched; however, it is turned on when bound to integrin α_v_β_3_ and CD13. In terms of precise targeting protocol, enduring staining effect and benignancy to living cells, the aggregation-induced emission-based TCNTP probe has distinct advantages over conventional dyes. As a proof-of-concept, the targeting ability of TCNTP towards integrin α_v_β_3_ and CD13 in living cells was monitored in real time in co-cultured MDA-MB-231 and A375 cells, and HT-1080 and A375 cells. The colocalization experiments using commercial nucleus imaging dye Hoechst 33258 reveal that TCNTP can serve as a fluorescence light-up nuclear penetrating dye with the aid of functional peptide and AIEgens. Different from Hoechst 33258, the harmless physical internalization processes and indelible cellular staining property guarantee its long-term tumor cell tracing ability. The fluorescence stains of TCNTP are carried to daughter cells with low toxicity and remain visible even after the tenth culture cycle. Moreover, its precisely targeted interaction with organelles may account for the leak-free marking inside cells. These notable features make TCNTP a potential vehicle for drug and nucleic acid delivery, thus extending the application scope of theranostics.
