# Supplementary material for: Dual-targeted peptide-conjugated multifunctional fluorescent probe with AIEgen for efficient nucleus-specific imaging and long-term tracing of cancer cells
Source: Chem Sci. 2017 Apr 19;8(6):4571–8. doi: 10.1039/c7sc00402h (PMC5471453; doi:10.1039/c7sc00402h)
Supplement: Supplementary file 1 [file SC-006-C7SC00402H-s001.pdf]

Electronic Supplementary Material (ESI) for Chemical Science.
This journal is © The Royal Society of Chemistry 2017

Electronic Supplementary Information

Dual-targeted peptide-conjugated multifunctional fluorescent probe with AIEgen for efficient nucleus-specific imaging and long-term tracing of cancer cells

Yong Cheng,[†,‡] Chunli Sun,[†] Xiaowen Ou,[†] Bifeng Liu,[‡] Xiaoding Lou,*[,†] Fan Xia,*[,†,‡]

[†]Hubei Key Laboratory of Bioinorganic Chemistry & Materia Medica, School of Chemistry and Chemical Engineering, Huazhong University of Science and Technology, Wuhan 430074, P. R. China.

[‡]National Engineering Research Center for Nanomedicine, Department of Biomedical Engineering, College of Life Science and Technology, Huazhong University of Science and Technology, Wuhan 430074, P. R. China

Email: louxiaoding@hust.edu.cn

xiafan@hust.edu.cn

Materials and methods.

Sodium ascorbate, copper bromide and Hoechst 33258 were purchased from Aladdin. TCNT was designed by ourselves and customized from GL Biochem Ltd. (Shanghai, China). The integrin $\alpha_v\beta_3$ and CD13 were purchased from R&D Systems Inc. Thrombin (TB), Haemoglobin (HGB), Trypsin (TP), Carcinoembryonic antigen (CEA), Albumin from bovine serum (BSA) 3-(4,5-dimethylthiazol-2-yl)-2,5-diphenyl-tetrazolium bromide (MTT) was purchased from Sigma-Aldrich. Breast cancer cells (MDA-MB-231), fibrosarcoma cells (HT-1080), human malignant melanoma cells (A375) and human lung fibroblast cells (HLF) were purchased from Boshide (Wuhan, China). All other reagents were obtained from commercial sources and used without further purification.

^1H and ^{13}C NMR spectra were measured on a Bruker ARX 400 NMR spectrometer using chlroform-d ($CDCl_3$-d) as solvent and tetramethylsilane (TMS) as internal reference. Splitting patterns are reported as s (single), d (doublet), t (triplet) and m (multiplet). High resolution mass spectra (HRMS) were recorded on a Bruker micrOTOF II mass spectrometer system operating in a MALDI-TOF mode. High performance liquid chromatography (HPLC) was performed by using Agilent 1000 for analytical HPLC and Wufeng LC100 for semi-preparative HPLC. The sample was dissolved in water solution or acetonitrile, applied on a Kromasil C18 column (10 μm, 250 × 4.6 mm) from Teknokroma, and eluted at 2 mL/min with a 40 min gradient from 40% to 95% solvent B, where solvent A is water (0.1% TFA solution) and solvent B is acetonitrile (0.1% TFA solution). All products were purified by HPLC to reach purity of 95%. UV-Vis absorption spectra were taken on an Agilent Cary 60 UV/Visible Spectrometer. All fluorescence measurements were performed on an Agilent Cary Eclipse Fluorescence Spectrophotometer. Confocal laser scanning microscopy images were obtained on a Fluoview FV1000 confocal laser scanning microscope (Olympus). MTT assay was obtained on an Infinite M200 PRO Microplate Reader (Tecan Austria).

Synthesis and Characterization

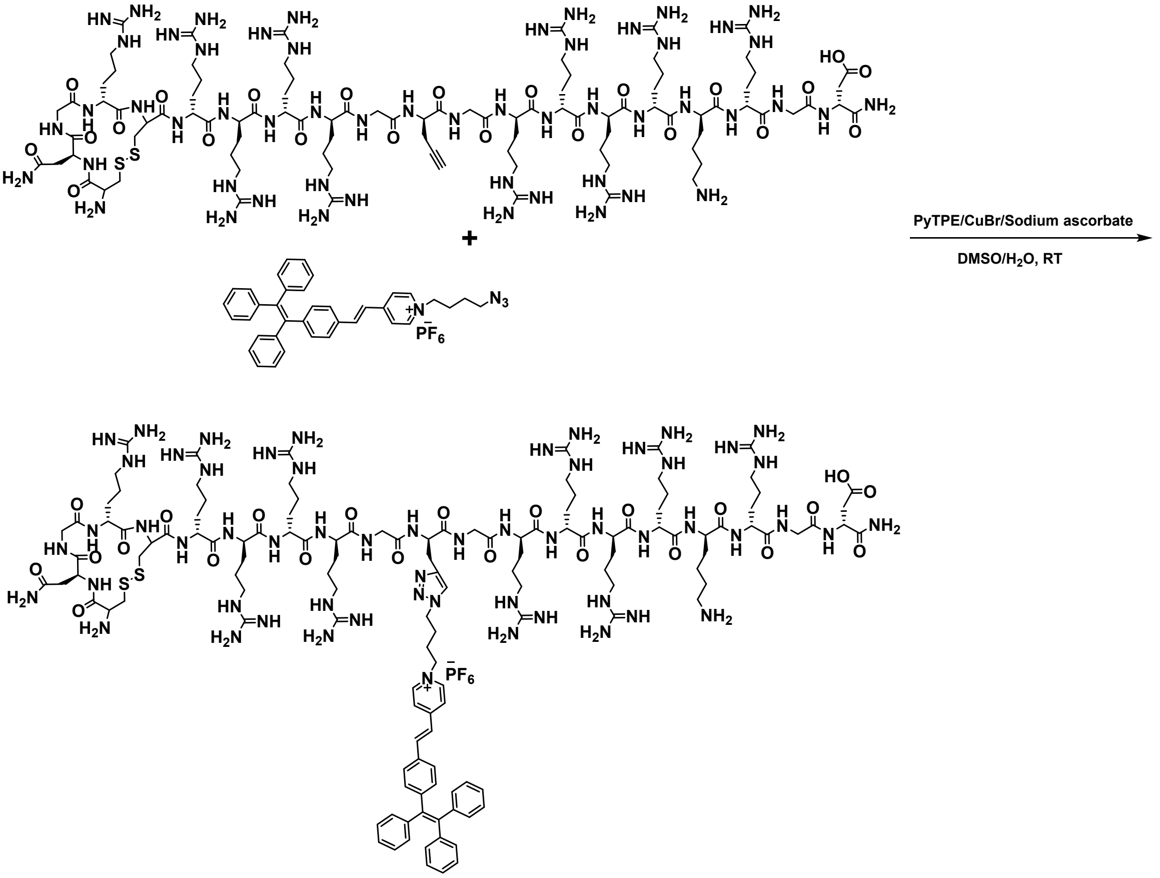

Scheme S1. The synthetic routes of TCNTP.

Synthesis of DOX-FCPPs-PyTPE (TCNTP).

The azide-containing tetraphenylethene (PyTPE, 11.0 mg, 20.0 μmol) was dissolved in DMSO (0.5 mL). The solution of the TCNT (10.0 mg, 4.1 μmol), ascorbic acid sodium salt (4.0 mg, 2.0 μmol) and copper (I) bromide (1.5 mg, 1 μmol) in 0.5 mL deionized water were added and the reaction mixture was shaken for 48 h at room temperature. The final product was purified by semi-preparative HPLC and obtained 10 mg of light yellow solid TCNTP (yield 76%). HRMS (ESI) m/z: $[M + 2H]^{2+}/2$ calcd for 1498.8072; found, 1498.8122. $[M + 3H]^{3+}/3$ calcd for 999.5407; found, 999.5478. $[M + 4H]^{4+}/4$ calcd for 749.9075; found,

749.9139. [M + 5H]$^{5+}$/5 calcd for 600.1276; found, 600.1340. [M + 3H]$^{6+}$/6 calcd for 500.2743; found, 500.4466. [M + 3H]$^{7+}$/7 calcd for 428.9505; found, 428.9555. [M + 3H]$^{8+}$/8 calcd for 375.4451; found, 375.4628.

Cell culture

MDA-MB-231 cells and A375 cells were cultured in Dulbecco's Modified Eagle Medium (DMEM); HT-1080 cells and HLF cells were cultured in minimum essential medium (MEM); the co-cultured cells were cultured in Dulbecco's Modified Eagle Medium (DMEM) with the same densities. All the cells were finally cultured with 10% fetal calf serum (FBS) and 1% penicillin streptomycin (PS, 10000 IU penicillin and 1000 μg/mL streptomycin, Multicell) in a culture flask at 37 °C in a humidified atmosphere containing 5% CO_2.

Incubation Living Cells with Probe or Drug.

For confocal laser scanning microscopy imaging, cells were seeded into cell culture dishes at a density of 2.0×10^4 in growth medium (DMEM or MEM supplemented with 10% FBS, 200 mL). After an overnight incubation, the cells were washed with phosphate-buffered saline (PBS, pH 7.2−7.4) for three times. A solution of the indicated probe in medium was then added, and the cells were incubated in a 5% CO_2 atmosphere at 37 °C for further usage. Especially, to avoid interference during co-localization with commercial dyes, Hoechst 33258 would subsequently add after the incubation of PyTPE or TCNTP. The supernatant was then discarded, and the cells were washed gently twice with PBS and immersed in growth medium prior to optical imaging.

Confocal laser scanning microscopy.

The fluorescence signals were detected by using a Fluoview FV1200 confocal laser scanning microscope (Olympus), equipped with a 60/1.42 numerical aperture oil-immersion objective lens. A 405 nm laser was chosen for the excitation of PyTPE and the emission was collected at 495–575 nm. A 405 nm laser was chosen for the excitation of Hoechst33258 and the emission was collected at 425–485 nm. Real-time confocal imaging was performed by the incubation system for microscopes (Tokai Hit,

Japan). All fluorescence images were analysed with FV10-ASW V4.0 Image software (Olympus). Three-dimensional map of cells were used 3D rendering by Imaris (Andor-Bitplane, Zurich). Co-localization analysis were used Image-Pro Plus 6.0.

Cytotoxicity assay.

The cytotoxic potential of PyTPE, Hoechst 33258 and TCNTP was assessed using the MTT assay. MDA-MB-231 cells, HT-1080 cells, A375cells and HLF cells were respectively treated with PyTPE (1 μM, 5 μM and 10 μM), Hoechst 33258 (1 μM, 5 μM and 10 μM) and TCNTP (1 μM, 5 μM and 10 μM) for 48 h in triplicate in a 96-well plate. The absorbance of MTT at 570 nm was recorded by the Infinite M200 PRO microplate reader.

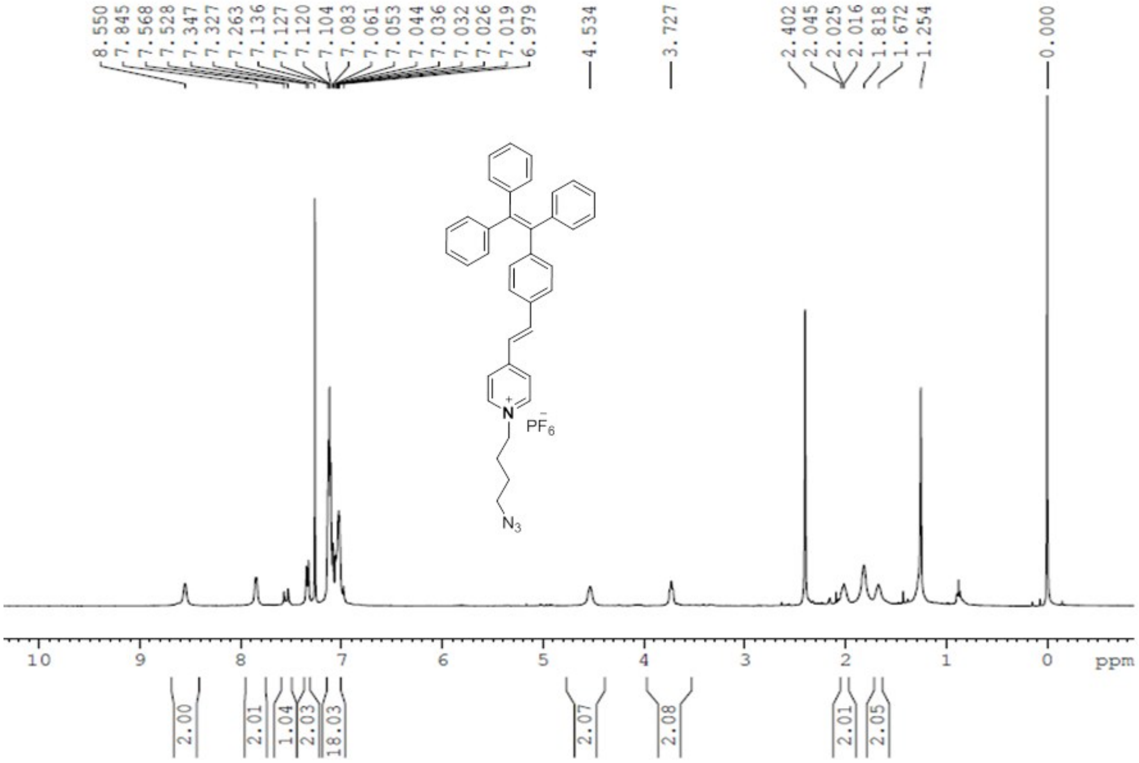

Fig. S1 ^{1}H-NMR spectra of compound PyTPE in $CDCl_3$.

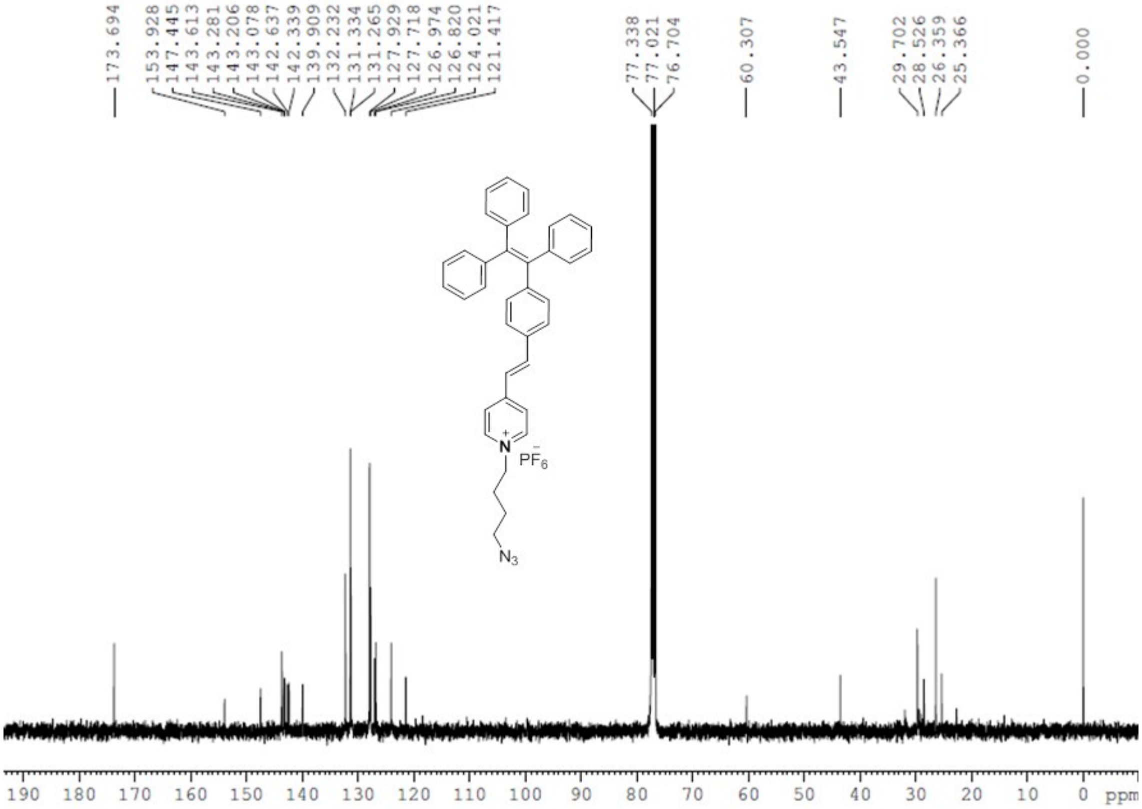

Fig. S2 ^{13}C-NMR spectra of compound PyTPE in $CDCl_3$.

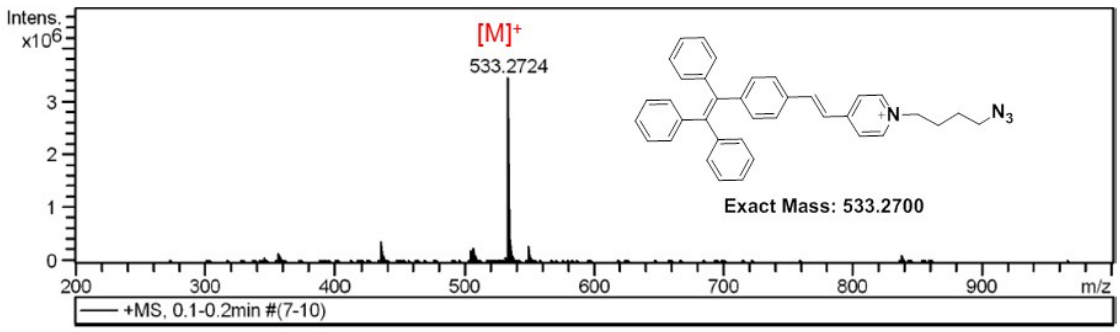

#	m/z	Res.	S/N	I	FWHM
1	533.2724	20522	5654.2	3450492	0.0260
2	534.2740	18464	2457.0	1503619	0.0289
3	535.2769	14144	672.9	412976	0.0378

Fig. S3 HRMS (MALDI-TOF) of compound PyTPE.

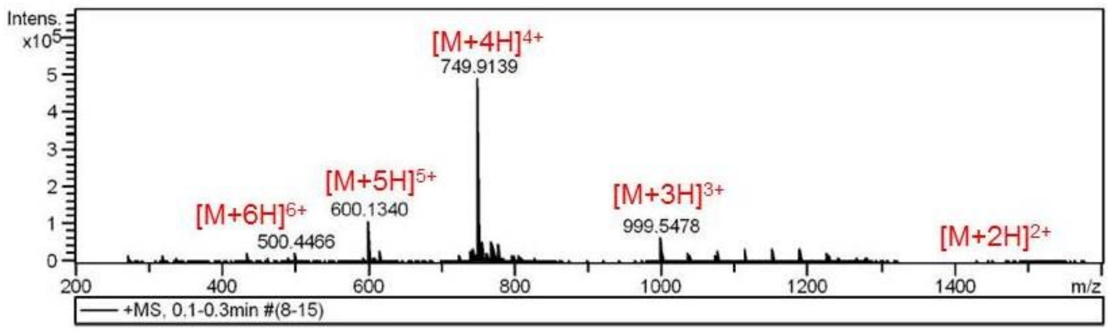

#	m/z	Res.	S/N	I	FWHM
1	375.4628	19573	8.2	1257	0.0192
2	428.9555	14865	32.3	6367	0.0289
3	500.4466	21660	86.2	22808	0.0231
4	600.1340	16580	250.5	109485	0.0362
5	749.9139	18694	700.6	491020	0.0401
6	999.5478	18823	194.3	64115	0.0531
7	1498.8122	20473	14.5	3254	0.0732

TCNTP	M	M+H	[M+2H]	[M+3H]	[M+4H]
molecular weight	2995.5987	2996.6065	2997.6144	2998.6222	2999.6300
m/q			1498.8072	999.5407	749.9075
	[M+5H]	[M+6H]	[M+7H]	[M+8H]	
molecular weight	3000.6378	3001.6457	3002.6535	3003.6613	
m/q	600.1276	500.2743	428.9505	375.4451	

Fig. S4 HRMS (MALDI-TOF) of compound TCNTP.

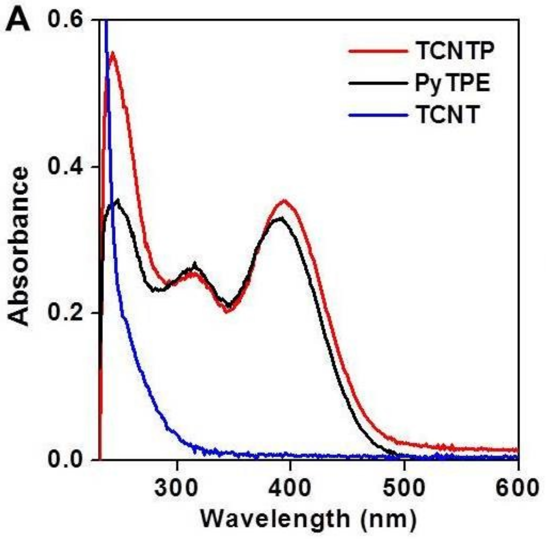

Fig. S5 (A) The UV-vis absorption spectra of TCNTP, PyTPE and TCNT; (B) Plot of I/I_0 versus different concentrations of TCNTP (1.0 μM, 5.0 μM, 10.0 μM, 25.0 μM, 50.0 μM and 100.0 μM) treated with (I) and without (I_0) $\alpha_v\beta_3$ (10 μg/mL), respectively.

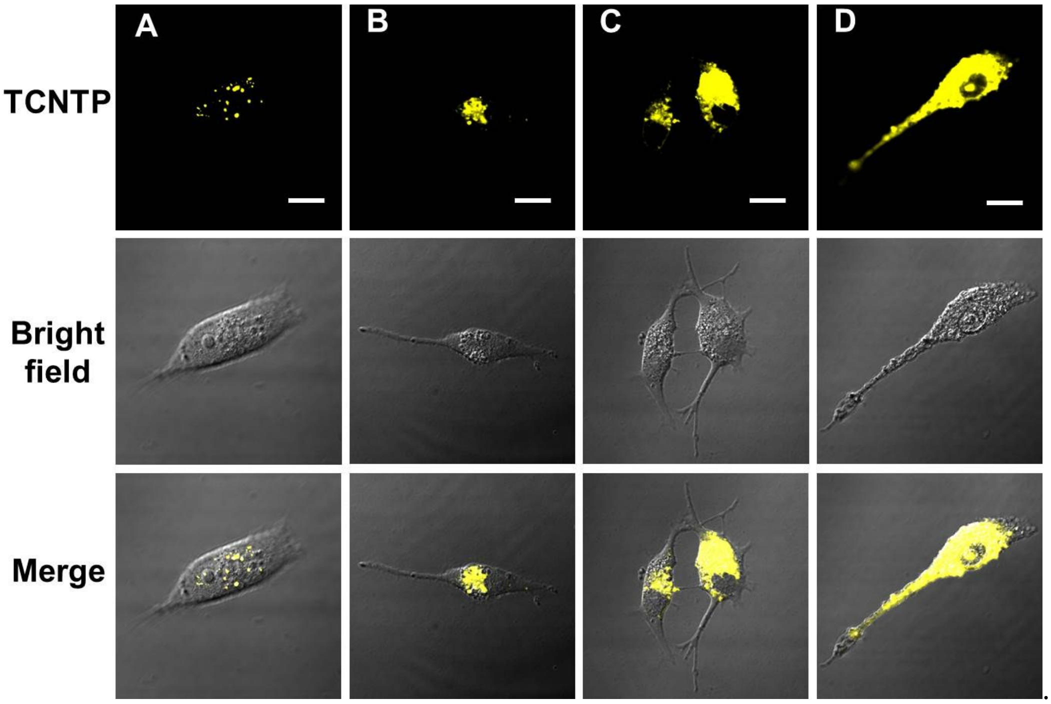

Fig. S6 Fluorescence images of MDA-MB-231 cells incubated with different concentrations of TCNTP (1.0 μM (A), 3.0 μM (B), 5.0 μM (C) and 10.0 μM (D)) for 4 h in cell culturing condition. The scale bar is 10 μm.

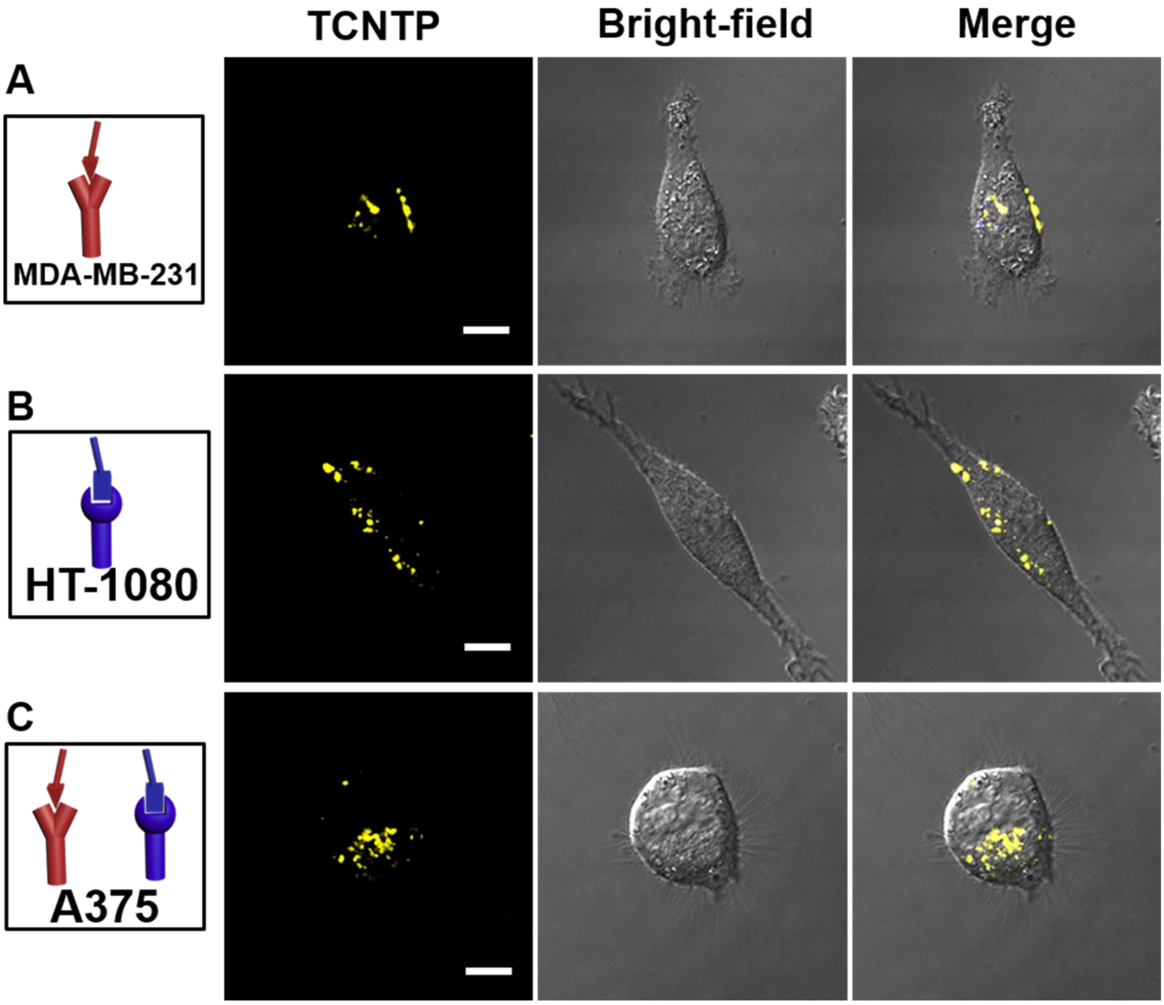

Fig. S7 In-situ and real-time fluorescence images (A) and its corresponding three-dimensional map (B) of co-cultured MDA-MB-231 cells (red ellipse) & A375 cells (green ellipse) incubated with 1 μM TCNTP for 25 min; (C) The average fluorescence intensity of MDA-MB-231 & A375 co-cultured cells incubated with 1.0 μM TCNTP for 25 min. Green bars represent the average fluorescence intensity of A375 cells; red bars represent the average fluorescence intensity of MDA-MB-231cells.

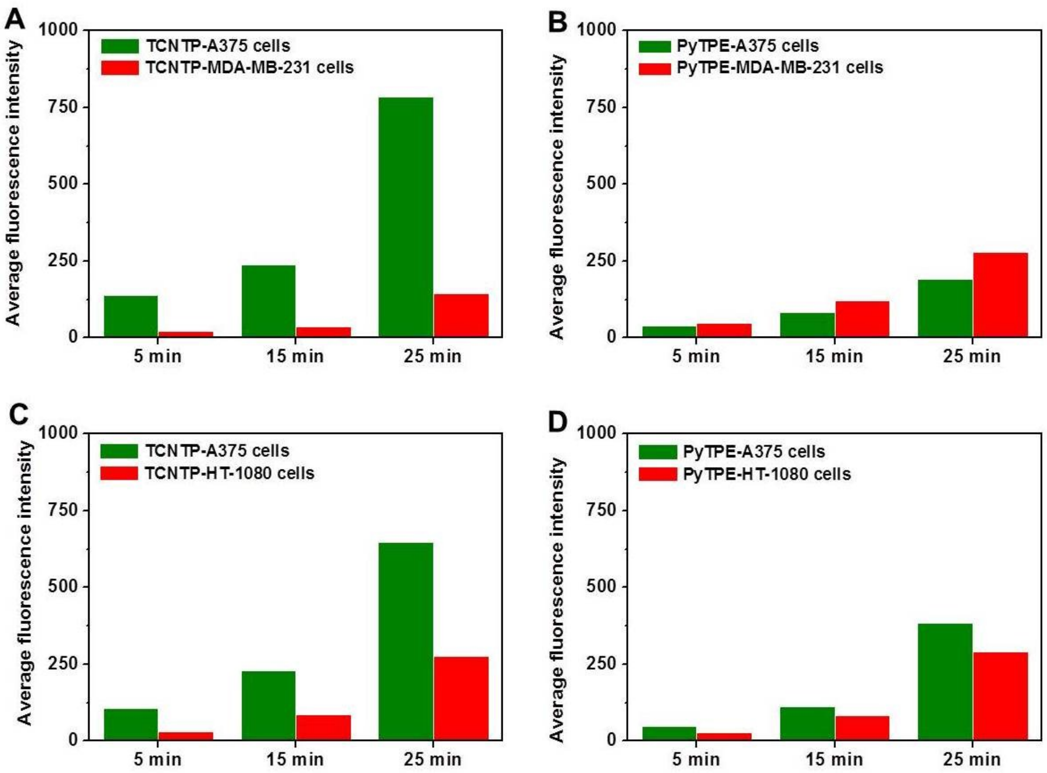

Fig. S8 The average fluorescence intensity of MDA-MB-231 & A375 co-cultured cells and HT-1080 & A375 co-cultured cells incubated with 3.0 μM TCNTP and PyTPE for 25 min, respectively. Green bars represent the average fluorescence intensity of A375 cells; red bars represent the average fluorescence intensity of MDA-MB-231cells or HT-1080 cells.

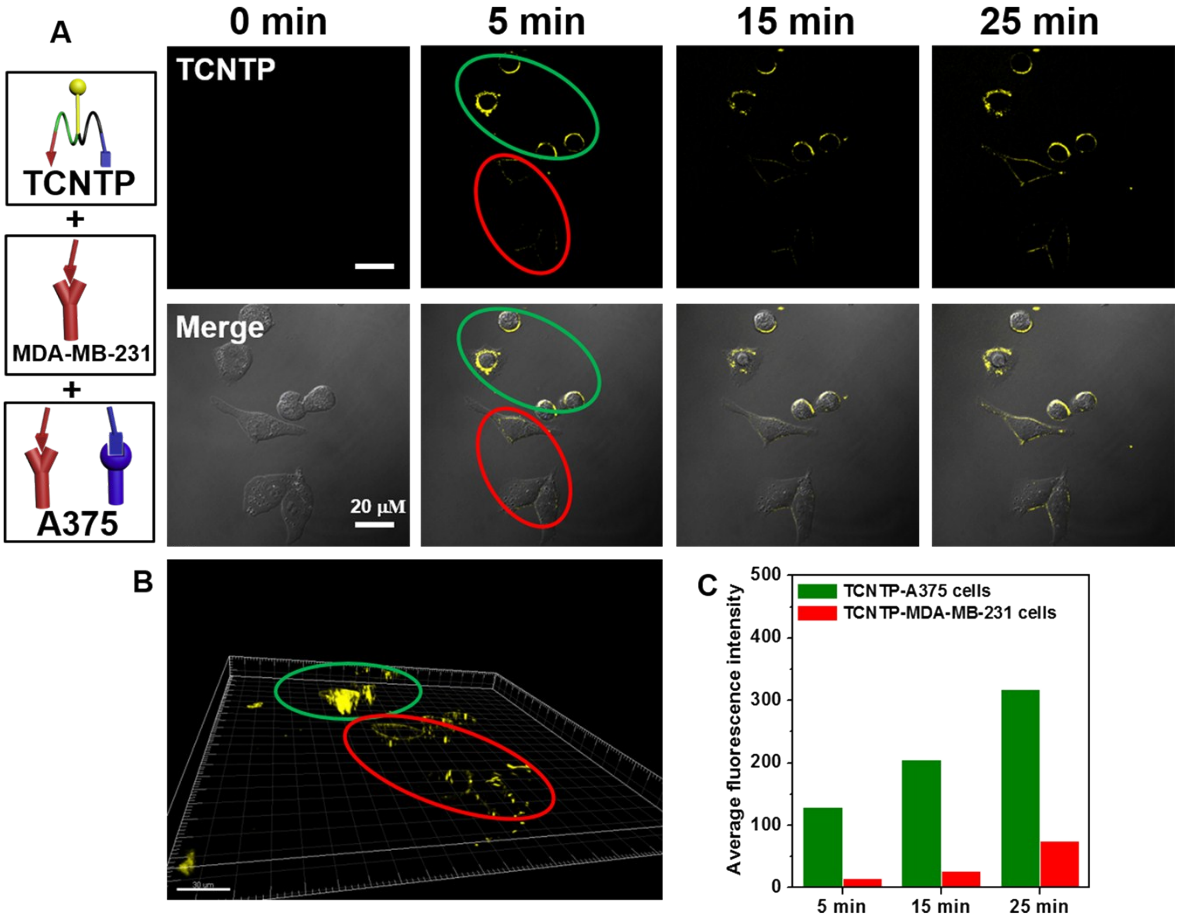

Fig. S9 Fluorescence images of MDA-MB-231 cells (A) HT-1080 cells (B) and A375 cells (C) treated with 1 μM TCNTP for 30 min in cell culturing condition. The scale bar: 10 μm.

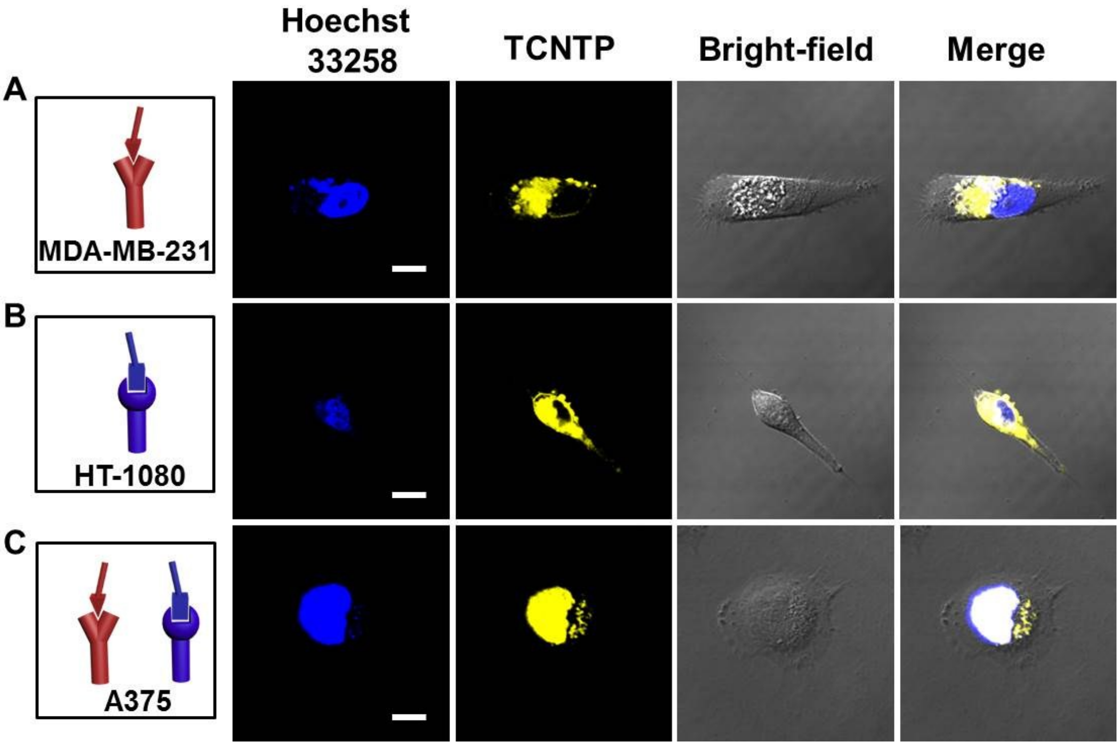

Fig. S10 Fluorescence images of MDA-MB-231 cells (A) HT-1080 cells (B) and A375 cells (C) treated with 3 μM TCNTP for 12 h and then 3 μM Hoechst33258 for 30 min in cell culturing condition. The white scale bar: 20 μm.

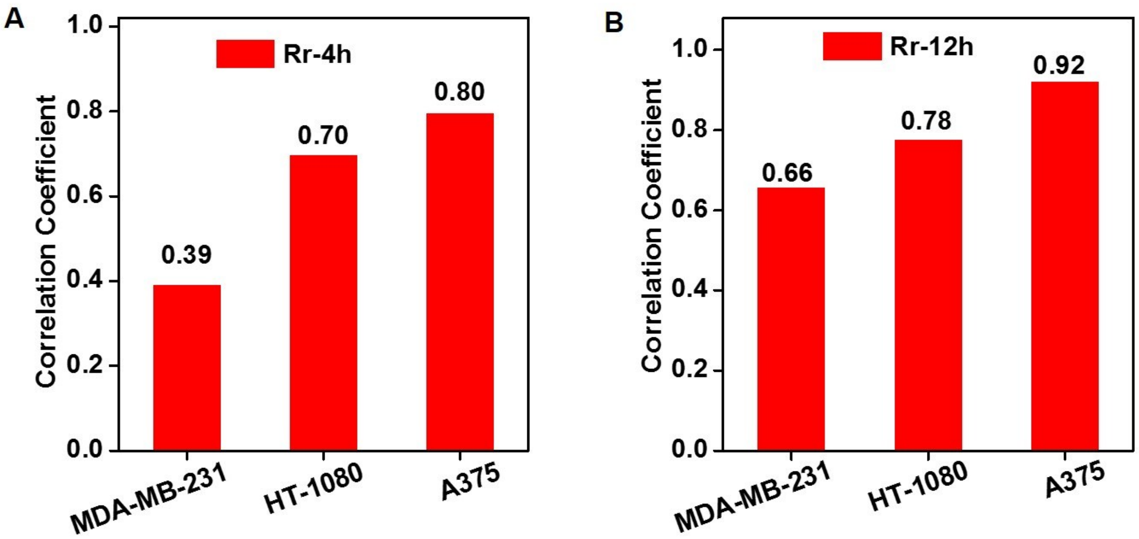

Fig. S11 The correlation coefficient of co-localization about MDA-MB-231 cells, HT-1080 cells and A375 cells treated with 3 μM Hoechst 33258 and TCNTP for 4 h (A) and 12 h (B) in cell culturing condition. Rr represents Pearson correlation coefficient.

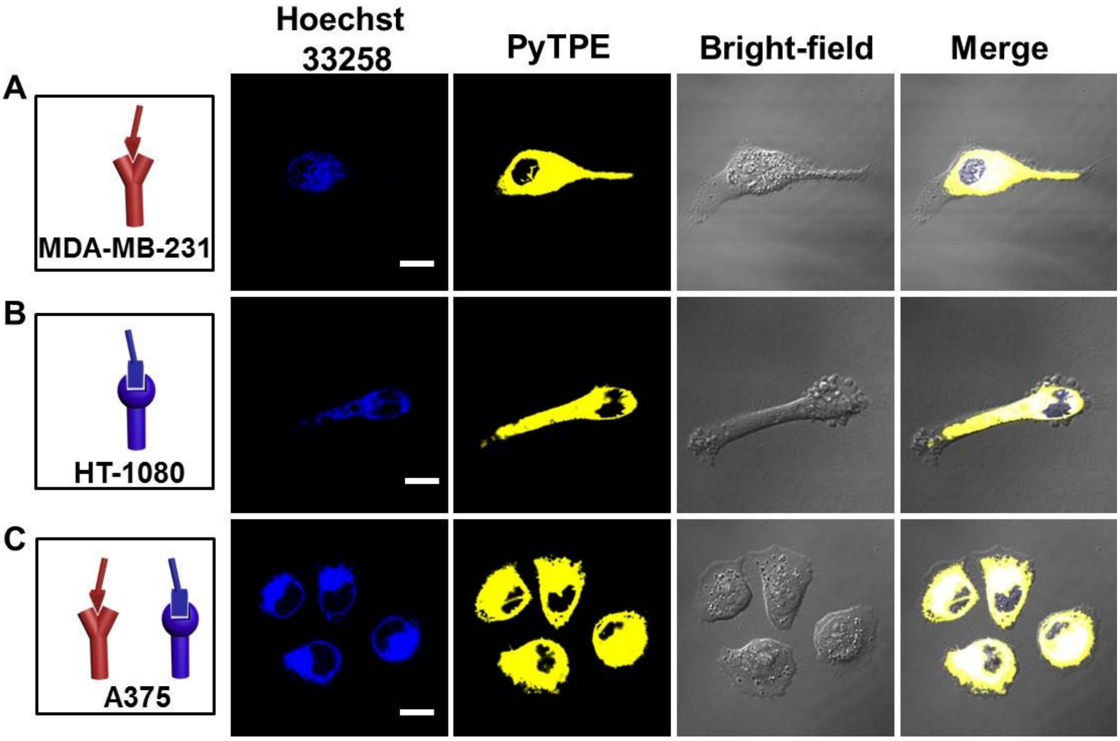

Fig. S12 Fluorescence images of MDA-MB-231 cells (A) HT-1080 cells (B) and A375 cells (C) treated with 3 μM PyTPE of each formulation for 12h and then 3 μM Hoechst33258 for 30 min in cell culturing condition. Scale bar: 20 μm.

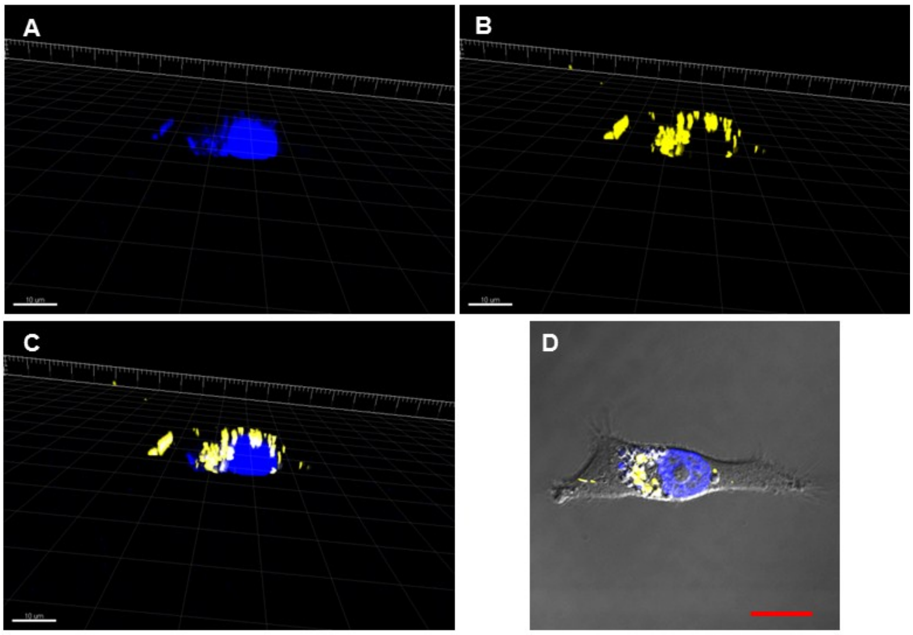

Fig. S13 Corresponding the three-dimensional map of MDA-MMB-231 cells for incubation with 3.0 μM TCNTP for 12h, and then 3 μM Hoechst33258 for 30 min. The white scale bar: 10 μm; the red scale bar: 20 μm.

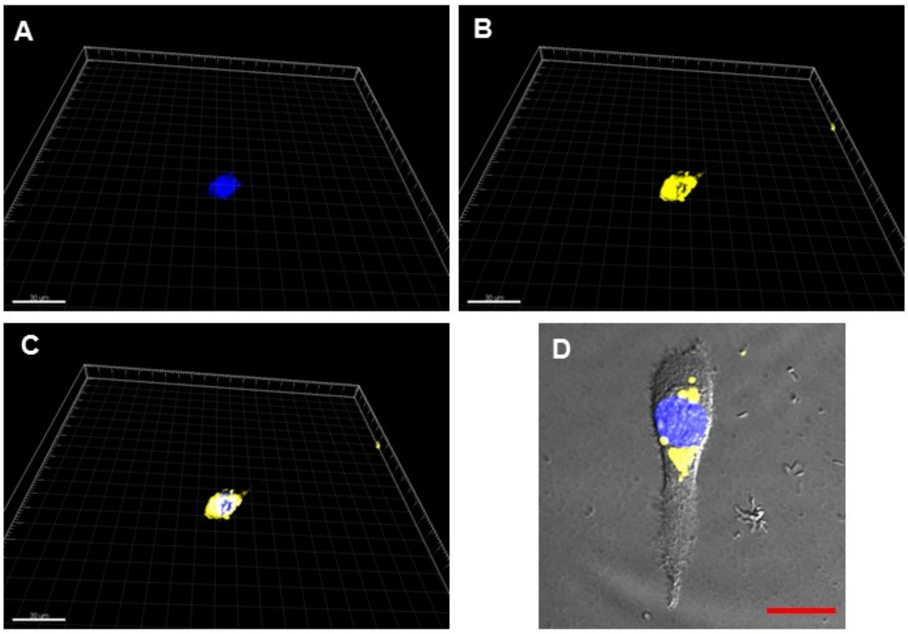

Fig. S14 Corresponding the three-dimensional map of HT-1080 cells for incubation with 3.0 μM TCNTP for 12h, and then 3 μM Hoechst 33258 for 30 min. The white scale bar: 30 μm; the red scale bar: 20 μm.

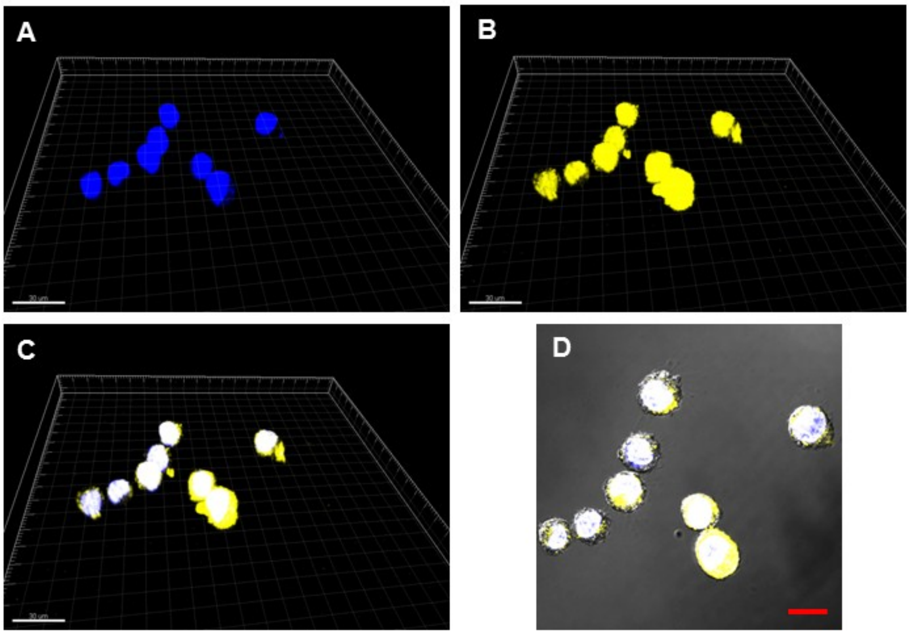

Fig. S15 Corresponding the three-dimensional map of A375 cells for incubation with 3.0 μM TCNTP for 12h, and then 3 μM Hoechst 33258 for 30 min. The white scale bar: 30 μm; the red scale bar: 20 μm.

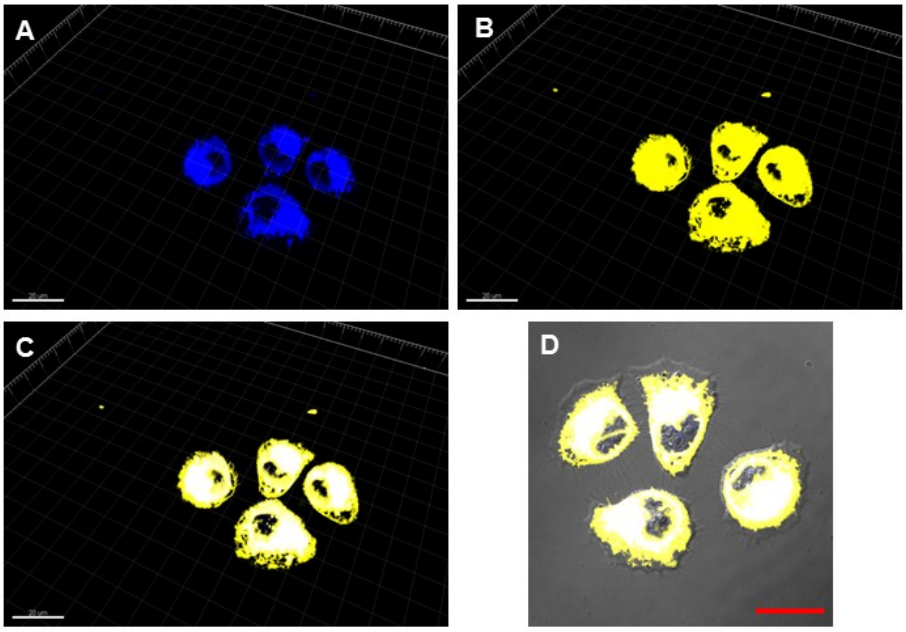

Fig. S16 Corresponding the three-dimensional map of A375 cells for incubation with 3.0 μM PyTPE for 12h, and then 3 μM Hoechst 33258 for 30 min. The white scale bar: 20 μm; the red scale bar: 20 μm.

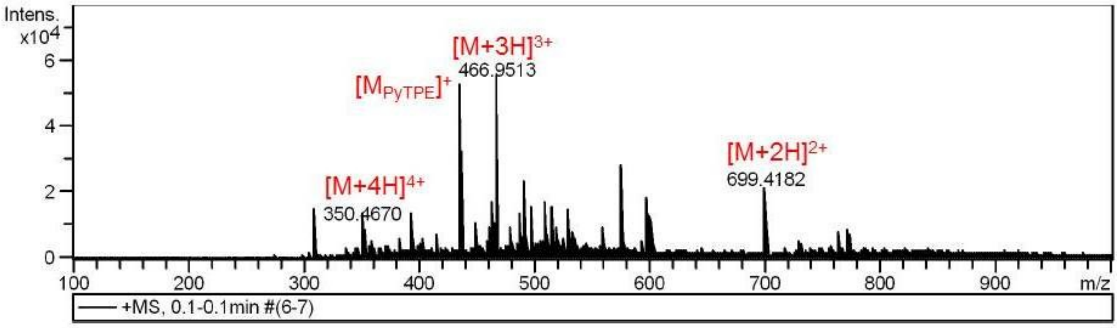

#	m/z	Res.	S/N	I	FWHM
1	350.4670	11690	24.5	9730	0.0300
2	466.9513	13345	82.1	52948	0.0350
3	699.4182	12823	32.3	20465	0.0545

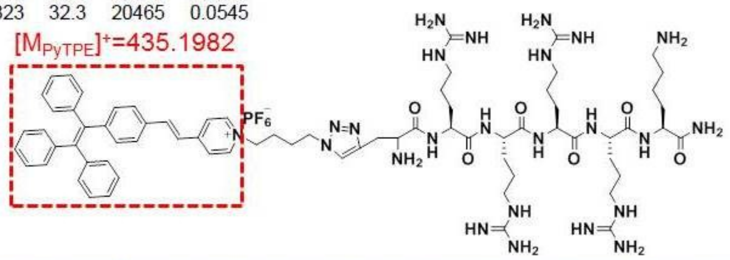

NLS-PyTPE	M	M+H	[M+2H]	[M+3H]	[M+4H]
molecular weight	1397.8330	1398.8409	1399.8487	1400.8565	1401.8643
m/q			699.9244	466.9522	350.4661

Fig. S17 HRMS (MALDI-TOF) of compound NLS-PyTPE.

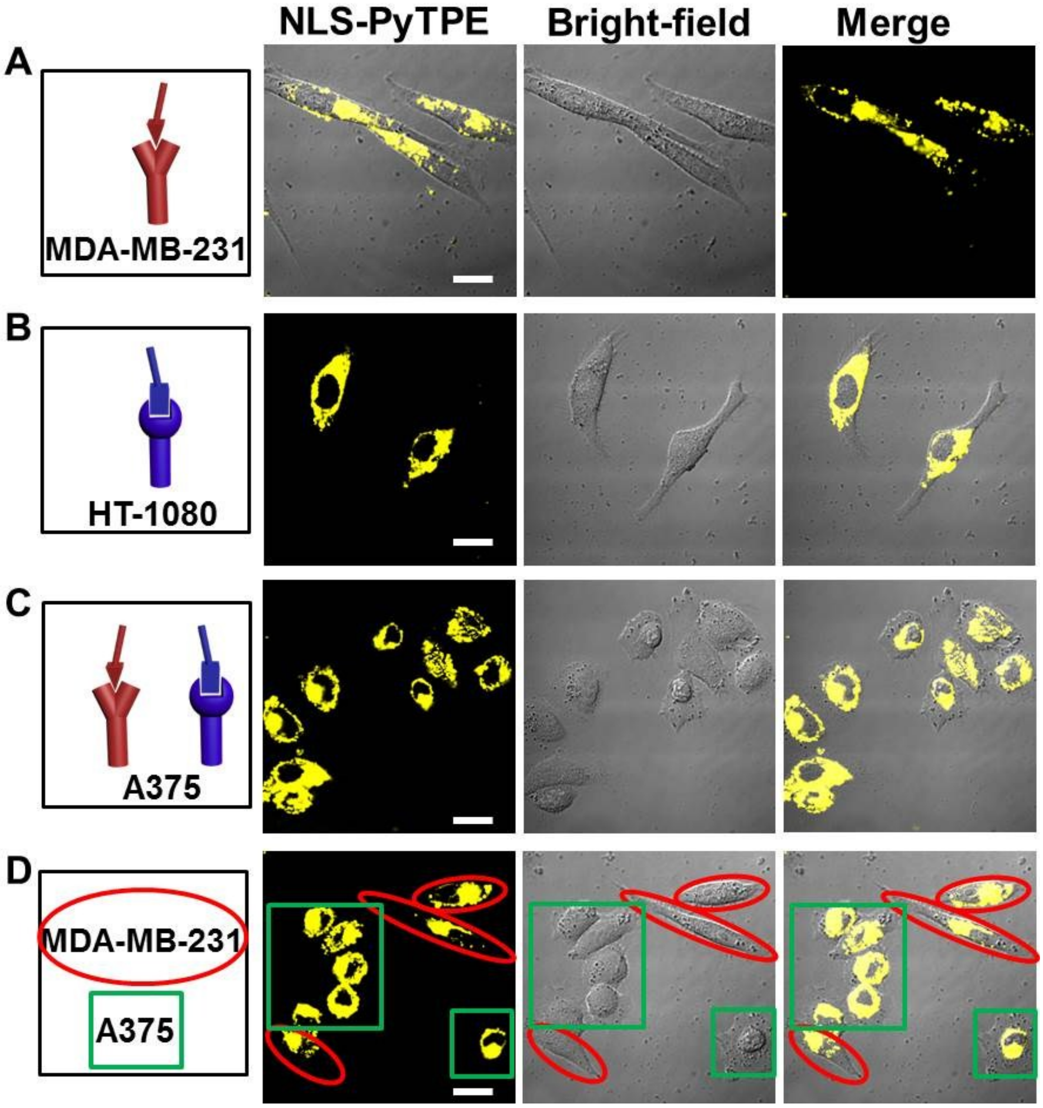

Fig. S18 Fluorescence images of MDA-MB-231 cells (A), HT-1080 cells (B), A375 cells (C) and MDA-MB-231 (red ellipse) & A375 (green square) co-cultured cells (D) treated with 3 μM NLS-PyTPE for 4 h in cell culturing condition. The white scale bar: 20 μm.

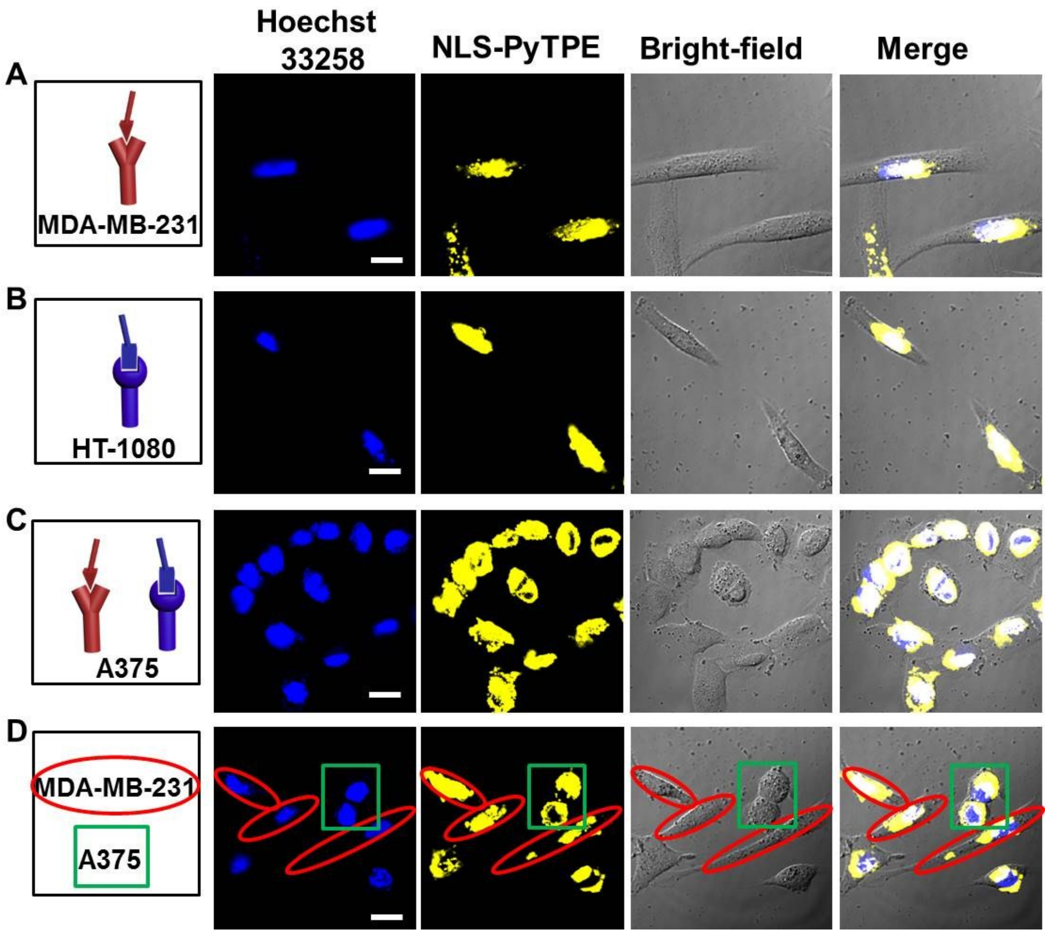

Fig. S19 Fluorescence images of MDA-MB-231 cells (A), HT-1080 cells (B), A375 cells (C) and MDA-MB-231 (red ellipse) & A375 (green square) co-cultured cells (D) treated with 3 μM NLS-PyTPE for 8 h, and then 3 μM Hoechst 33258 for 30 min in cell culturing condition. The white scale bar: 20 μm.

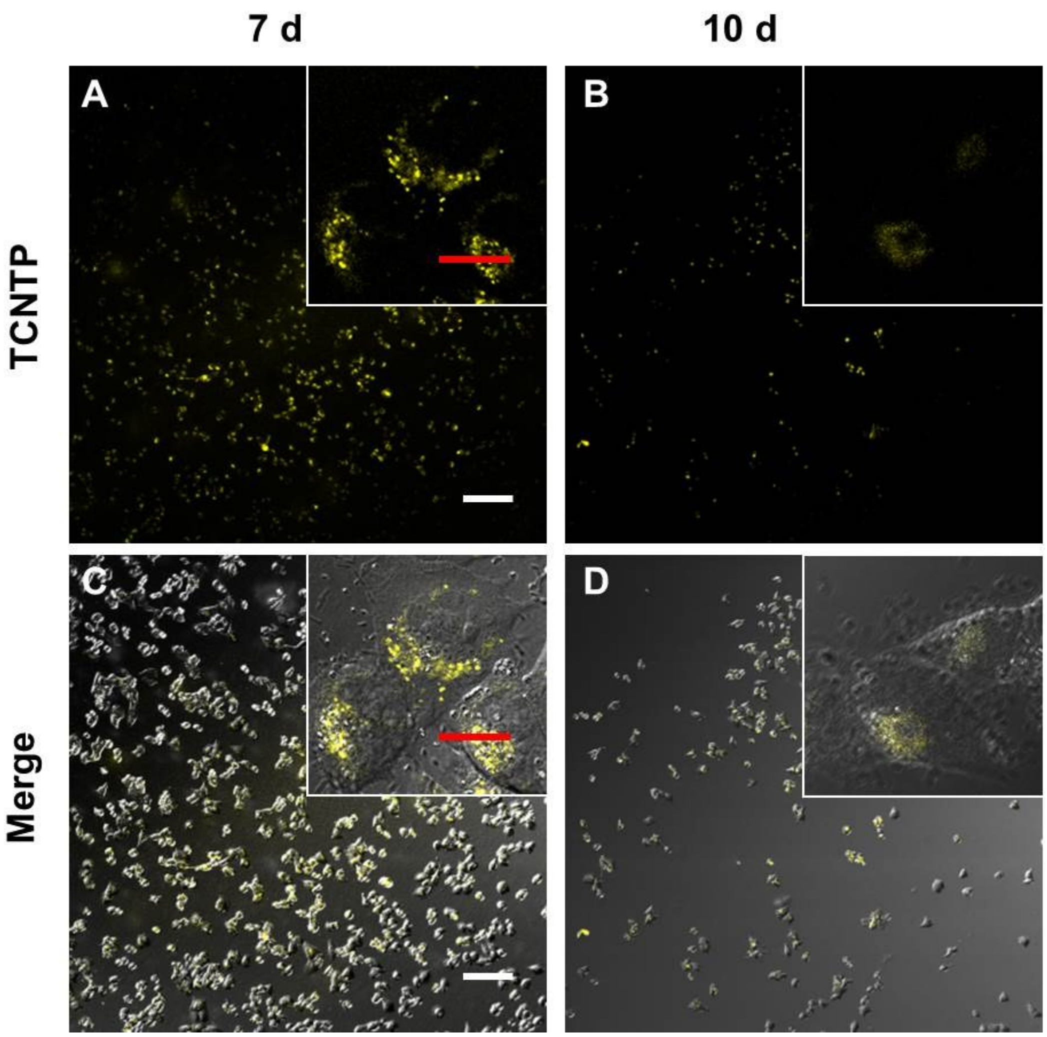

Fig. S20 Fluorescence images of A375 cells treated with 3 μM TCNTP and Hoechst 33258 for 4h with further incubation in 7d (A, C) and 10d (B, D), respectively. The white scale bar: 100 μm, the red scale bar: 10 μm.

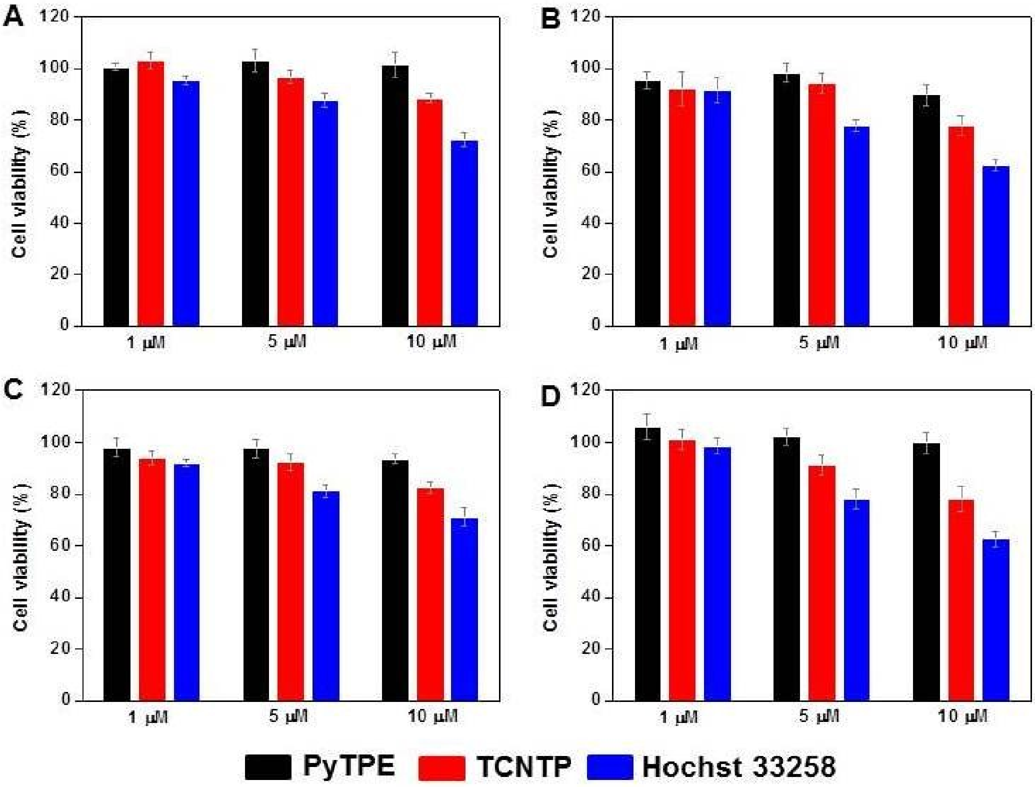

Fig. S21 Metabolic viability of (A) MDA-MB-231 cells, (B) HT-1080 cells, (C) A375 cells and (D) HLF cells after incubation with PyTPE and TCNTP at concentrations of 1 μM, 5 μM and 10 μM for 48 h. Black bars represent PyTPE, red bars represent TCNTP and blue bars represent Hoechst 33258.

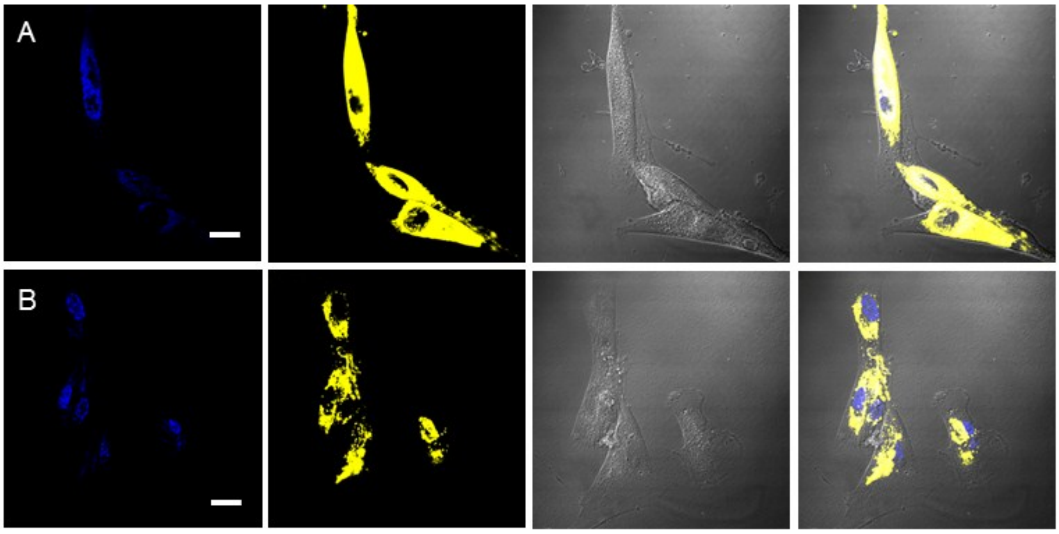

Fig. S22 Fluorescence images of HLF cells treated with 3 μM PyTPE (A) and TCNTP (B) with Hoechst 33258 of each formulation for 12h in cell culturing condition. The white scale bar: 20 μm.
